# Dynamic Migratory Strategies and Foraging Habitats of Southern Right Whales Revealed by Satellite Telemetry

**DOI:** 10.1002/ece3.73975

**Published:** 2026-07-06

**Authors:** Matthew Germishuizen, Alexandre N. Zerbini, Amy Kennedy, Marcello Vichi, Christopher Wilkinson, Els Vermeulen

**Affiliations:** ^1^ Mammal Research Institute Whale Unit, Faculty of Natural and Agricultural Sciences University of Pretoria Pretoria South Africa; ^2^ Cooperative Institute for Climate, Ocean, and Ecosystem Research (CICOES), University of Washington Seattle Washington USA; ^3^ Marine Mammal Laboratory, Alaska Fisheries Science Center National Marine Fisheries Service, National Oceanic and Atmospheric Administration Seattle Washington USA; ^4^ Instituto Aqualie Juiz de Fora Minas Gerais Brazil; ^5^ Marine Ecology and Telemetry Research Seabeck Washington USA; ^6^ Department of Oceanography University of Cape Town Cape Town South Africa; ^7^ Marine and Antarctic Research Centre for Innovation and Sustainability (MARIS), University of Cape Town Cape Town South Africa

**Keywords:** climate, feeding ecology, habitat modelling, random forest, Southern Ocean

## Abstract

Understanding the migratory and foraging behaviour of wide‐ranging marine predators is crucial for effective conservation planning, particularly in capital breeders where foraging success directly influences reproductive success and population dynamics. For South Africa's southern right whale (SRWs), reproductive success has declined over recent decades, yet contemporary offshore migratory routes and foraging habitats remain poorly known. Here, satellite‐linked tags deployed on 34 adult SRWs between 2021 and 2024 were used to investigate migratory strategies and identify environmental correlates of offshore area‐restricted search (ARS) behaviour. ARS locations derived from a movement‐persistence model were linked to environmental covariates using a random‐forest habitat model to identify predictors of foraging behaviour, map suitable offshore foraging habitat, and predict habitat suitability across the wider Southern Ocean foraging range of the South African population. Whales ranged widely across the South Atlantic and southwest Indian Oceans, concentrating foraging effort within the Antarctic Circumpolar Current (ACC), the high‐latitude seasonal ice zone, around the Crozet Islands, and within the Benguela upwelling system. Seven individuals (21%) undertook trans‐Atlantic migrations to foraging grounds shared with the SRW population of the Southwest Atlantic, demonstrating basin‐scale connectivity between populations. Random forest analyses identified environmental covariates associated with ARS behaviour across multiple oceanographic regions with high predictive performance (AUC = 0.90). Predicted habitat suitability highlighted suitable offshore foraging habitat associated with frontal systems, shelf‐associated habitats, upwelling regions, and high‐latitude seasonal ice habitats across the Southern Ocean. These findings provide the first environmentally explicit habitat‐modelling assessment of contemporary offshore foraging habitat for the South African SRW population. Results show this population exhibits diverse migratory routes and foraging behaviours, relying on a wide range of oceanographic habitats that may facilitate behavioural responses and plasticity to variability in foraging conditions across the Southern Ocean.

## Graphical abstract

Satellite‐linked tags on 34 southern right whales revealed basin‐scale movements and foraging concentrated in the ACC, Crozet Islands, Benguela system, and the highly variable seasonal ice zone. Environmental modelling revealed environmental drivers behind foraging decisions, and identified key ecosystems. These results provide a contemporary view of migratory strategies and pinpoint climate‐sensitive habitats requiring targeted conservation monitoring.

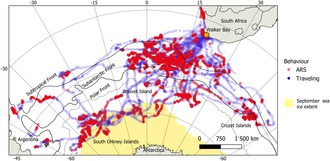

## 1 Introduction

Migratory marine animals are under threat due to mounting anthropogenic stressors in their environments. Globally, long‐term monitoring of southern right whale (SRW) populations is revealing lengthening calving intervals (e.g., Vermeulen et al. 2025; O'Shannessy et al. 2025), slowed population growth rates (Crespo et al. 2019; Grundlehner et al. 2025), altered foraging ecology (Derville et al. 2023), and reduced body condition (Vermeulen, Thavar, et al. 2023). Several studies have further highlighted the sensitivity of SRWs to climate variability (Seyboth et al. 2016; Agrelo et al. 2021), with some directly linking declining reproductive success to environmental alterations in offshore foraging grounds (Germishuizen et al. 2024; Charlton et al. 2026). These findings suggest that changes in Southern Ocean ecosystems may be influencing prey availability and foraging success across multiple SRW populations. This has created an urgency in furthering our understanding of SRW foraging habitats, particularly under the altered climate regimes that have developed across the Southern Ocean during the early 21st century (Schroeter et al. 2023; Ichii et al. 2023).

Due to limited long‐term data on zooplankton abundance and distribution, studies investigating climate‐change impacts on marine predators have largely relied on broad ecosystem indicators, such as sea‐surface temperature, chlorophyll concentration, and sea‐ice dynamics, to infer potential changes in prey availability (Tulloch et al. 2018). Consequently, much of the current understanding of how environmental variability influences baleen whale populations is based on inferred relationships between oceanographic change and prey dynamics, rather than direct examination of whale behavioural responses to prey availability. Improving understanding of how these environmental proxies relate to whale behaviour is therefore critical for interpreting ecological responses to climate change and for more accurately characterising suitable foraging habitat. Earlier efforts to characterise SRW feeding habitats using historical whaling records and climatological environmental covariates successfully identified broad oceanographic features associated with whale distribution, particularly frontal systems (Carman et al. 2019). However, these approaches provide only limited insight into the dynamic environmental processes shaping foraging behaviour at ecologically relevant spatial and temporal scales. Satellite telemetry, particularly when combined with behavioural and habitat‐modelling approaches, provides an opportunity to directly assess how variability in these environmental proxies influences whale behaviour by linking movement and behavioural states to the oceanographic conditions encountered by individuals (Reisinger et al. 2021). This enables finer‐scale examination of behavioural responses to environmental variability and oceanographic covariates, improves identification of environmentally suitable feeding habitats, and helps resolve the oceanographic mechanisms that generate favourable foraging conditions.

Over the past two decades, satellite telemetry programmes have substantially advanced understanding of SRW migratory connectivity and broad‐scale movement patterns across ocean basins. Telemetry studies have now been conducted across most major SRW calving grounds (Carroll et al. 2011; Mackay et al. 2020; Vermeulen, Germishuizen, et al. 2023; Watson et al. 2021; Zerbini et al. 2006, 2025), as well as at sub‐polar feeding and socialising grounds (Kennedy et al. 2023; Weir et al. 2024). Collectively, these studies have revealed extensive offshore migrations, shared feeding grounds between populations, and broad use of frontal systems and high‐latitude habitats. However, most telemetry‐based studies of SRWs have remained primarily descriptive, focusing on migratory routes, seasonal distributions, and broad‐scale habitat use, with comparatively limited attempts to explicitly quantify the environmental drivers underlying foraging behaviour. More recent work has begun moving beyond descriptive movement analyses towards examining behavioural responses to mesoscale oceanographic variability, including frontal systems and eddies (e.g., Riekkola et al. 2025).

For the South African population in particular, contemporary offshore migratory behaviour and foraging habitat remain comparatively poorly resolved. Existing understanding is still based largely on historical whaling records and a limited number of satellite telemetry tracks collected more than two decades ago (Mate et al. 2011; Tormosov et al. 1998). Satellite telemetry deployments during 2001–2002, although limited to only five offshore movements, suggested that high‐latitude feeding near Bouvet Island and the seasonal ice zone represented a dominant migratory strategy for the South African population, while one individual remained largely within the mid‐latitudes (Mate et al. 2011). In this study, satellite telemetry data from 34 SRWs tagged off South Africa between 2021 and 2024 were analysed to characterise contemporary migratory routes and behaviour. Specifically, this study aimed to: (1) describe the large‐scale migratory movements and seasonal distribution of South African SRWs; (2) determine the environmental variables most strongly associated with foraging behaviour using a random forest habitat model; and (3) predict the spatial and temporal distribution of suitable foraging habitat across the broader Southern Ocean domain.

## 2 Methods

### 2.1 Fieldwork

Boat‐based satellite tagging operations were carried out in Walker Bay (34.41° S, 19.27° E) on the southwestern coast of South Africa during October 2021, 2022, 2023 and 2024. During this period, fully integrated, consolidated Argos satellite‐linked tags were deployed on adult SRWs; Wildlife Computers, Redmond, WA; https://www.wildlifecomputers.com, including 32 SPOT‐372 and 2 SPLASH10‐373 tags. Tagging was conducted opportunistically on adult whales encountered in the study area, without targeting specific sex ratios or reproductive classes; however, individuals displaying visibly poor body condition, signs of injury, or apparent illness were not selected for tagging.

Of the 34 tags deployed on individual adult whales, only one was sexed as male. The remaining individuals were cows accompanied by calves and were therefore assumed to be female; 10 of these were also genetically confirmed as female. Information on each deployment can be found in Table S1, and individual tracks for each deployment year are presented in Figures [Link], [Link], [Link]. The average transmission duration was 209 ± 117 days (range = 5–491 days). Movements spanned latitudes from approximately 20.3° S to 63.4° S, and longitudes between 63.7° W and 58.3° E, while track lengths ranged from 208 km to 21,294 km.

Deployments of the tags were performed from a platform mounted on a 6 m rigid‐inflatable boat. All tags were deployed by an experienced tagger using a custom‐modified pneumatic rifle (Heide‐Jørgensen et al. 2001), at a distance of 3–5 m from the whales. Tags were deployed individually and attached to a delivery carrier, which detached upon successful implantation and was subsequently retrieved for reuse in future deployments. Biopsy samples were collected at the time of tagging for sexing using a Barnett Panzer V (150 lb. draw) crossbow. Subsequently, the tagged whale was monitored from a distance greater than 500 m for up to 30 min to capture post‐deployment images of the tag site.

### 2.2 Data Analysis

All analyses were performed in the R language environment (version 4.4.1, R Core Team 2024).

### 2.3 Movement Model

Satellite tags communicate with the ARGOS satellite system during periods when animals surface, with the transmission frequency determined by the combination of surfacing behaviour and satellite overpass availability. Location intervals are therefore generally irregular and can display substantial variability between individuals. Positions are assigned standard ARGOS location classes (LC 3, 2, 1, 0, A, B, Z), which represent decreasing positional accuracy, with LC Z representing invalid locations. High‐quality locations (LC 3–1) are generally obtained intermittently and are often interspersed with lower‐quality estimates, particularly at high latitudes where satellite coverage may be reduced. Coverage in the Southern Ocean can be variable due to satellite geometry and animal surfacing behaviour, occasionally resulting in multi‐hour to multi‐day gaps in transmission. Consequently, the effective spatial resolution of ARGOS‐derived tracks depends on transmission frequency and typically represents movements over spatial scales of several kilometres between successive fixes.

Prior to fitting the movement model, satellite telemetry tracks were first prepared by removing duplicate timestamps, filtering out low‐quality location estimates (e.g., Argos LC ‘Z’), and excluding implausible movements based on a speed threshold of 5 m/s. This follows standard Argos‐filtering procedures for marine mammals (Freitas et al. 2008), and applications to SRWs and humpback whales ( *Megaptera novaeangliae* ) which have used similar swim speed thresholds (Reisinger et al. 2021; Kennedy et al. 2023). Tracks were segmented if time gaps between consecutive locations exceeded 12 h, and only those with at least 50 locations were retained for analysis. This segmentation resulted in 139 independent track segments. The movement persistence (mp) model from the aniMotum R package (version 1.2–14, Jonsen et al. 2023) was applied using a 12‐h time step. The model is applied to regularise time steps and estimate behavioural tendencies. This time interval was selected after testing alternative intervals (6, 24, and 48 h) and was found to provide optimal model performance (Jonsen et al. 2023). Model convergence was assessed using the diagnostic output provided by the fitting routine, where convergence indicates that the optimisation procedure successfully reached a stable solution for the estimated movement parameters. Of the 139 fitted segments, 133 converged successfully and were retained for behavioural classification, while six segments that failed to converge were excluded from further analysis. In addition to convergence status, overall model stability was evaluated using the Hessian diagnostic provided by aniMotum. A positive‐definite Hessian indicates that the fitted solution is statistically stable and that the estimated parameters are reliable. All retained models returned a positive‐definite Hessian, providing additional confidence in the robustness of the behavioural estimates derived from the model.

The movement persistence model estimates a continuous behavioural parameter (*γ*) that quantifies the degree of autocorrelation in speed and direction between successive time steps. Values approaching 1 indicate highly persistent, directed movement, whereas values closer to 0 reflect reduced persistence characterised by shorter steps and greater turning variability. Area‐restricted search (ARS) therefore corresponds to periods of low movement persistence derived from this model‐based estimate of directional autocorrelation. A threshold of *γ* = 0.5 was used to differentiate between ARS and travelling behaviour. This threshold represents a common and interpretable cut‐off in movement ecology, with lower values (*γ* < 0.5) reflecting more tortuous and localised movement indicative of foraging or ARS, and higher values (*γ* > 0.5) corresponding to more directed, persistent travel (Florko et al. 2023; Shuert et al. 2023). Although ARS behaviour may also reflect social interactions, resting, or responses to complex environmental conditions, offshore ARS behaviour in this study was assumed to primarily represent foraging activity, consistent with previous telemetry studies of baleen whales in productive feeding habitats. As the precise value can vary across species and contexts, alternative movement‐persistence thresholds were examined to ensure the robustness of behavioural classification. These included using the median *γ* value as a binary threshold (e.g., Thums et al. 2022), as well as an upper–lower quartile approach in which low *γ* values (e.g., < 0.4) were classified as foraging, high *γ* values (e.g., > 0.6) as transiting, and intermediate values treated as mixed behaviour (Lydersen et al. 2020). These were used to confirm that the choice of *γ* cut‐off did not materially influence the results. Ultimately, it was determined that 0.5 provided a balanced, biologically meaningful division that avoids overly conservative or liberal classification of foraging. Model fit was assessed using one‐step‐ahead residual diagnostics on a representative track, evaluating the distribution and autocorrelation of residuals to confirm model adequacy. A description of the model diagnostics from the movement persistence model can be found in the Supporting Information.

### 2.4 Mean Latitude

To characterise the seasonal cycle in whale migratory behaviour, the mean and standard deviation of latitude were calculated for each Julian day across all individuals. October was excluded from the analysis to avoid artificially inflated variance associated with new tag deployments.

### 2.5 Track Length

Track length was calculated after fitting the movement persistence model, using the modelled location estimates for each individual track segment. Total track length was derived as the cumulative sum of great‐circle distances between successive locations along the track. Where substantial temporal gaps occurred between consecutive positions, straight‐line segments were used to connect these points. As a result, estimated track length represents the minimum distance travelled, as potential fine‐scale movements within data gaps could not be resolved.

### 2.6 Sexing

Eleven of the 34 tagged individuals were genetically sexed, while the remainder were sexed based on calf association, with individuals accompanied by a calf assumed to be female. Molecular sexing of individuals was completed by amplification of regions of the SRY and Zinc finger (ZFX) genes using a standard polymerase chain reaction (PCR) procedure. Primers used for this amplification were Y53‐3C and Y53‐3D (SRY) and P1‐5EZ and P2‐3EZ (ZFX). As males amplify the SRY region (located on the Y‐chromosome), they displayed two amplified bands, whereas females (with two X‐chromosomes) showed only one band.

### 2.7 Environmental Variables

Details on the seven environmental covariates extracted for each whale location are summarised in Table 1. To characterise the offshore environment used by SRWs, six dynamic environmental covariates were considered: SST, sea surface salinity (SSS), sea surface height (SSH), mixed layer depth (MLD), summer chlorophyll‐a concentration (CHL), and distance from the 15% September sea ice edge contour of the preceding winter (ICE). Environmental variables were matched to whale locations in a date‐specific manner. For datasets available at daily resolution (e.g., SST, SSH), values corresponding to the exact date of each whale position were extracted. For weekly or monthly products (e.g., SSS, MLD, CHL), the corresponding week or month for the specific year of the whale location was used (e.g., January 2019 values were extracted for whale positions in January 2019). Monthly climatologies were not used unless explicitly stated. Environmental fields were extracted at their native spatial resolution.

**TABLE 1:** Environmental variables extracted for each whale location.

Variable	Source	Resolution	Temporal resolution	Notes/description	DOI/website	Ecological significance
Bathymetry (BATHY; m)	GEBCO 2023	~15 arc‐second (500 m)	Static	Global terrain model for ocean and land.	https://www.gebco.net/data‐products/gridded‐bathymetry‐data	(Bestley et al. 2019; Garrigue et al. 2015; Harrison et al. 2020)
Chlorophyll (CHL; mg.m^−3^)	Aqua MODIS (NASA OceanColor)	4 km	Monthly	Level 3 mapped chlorophyll‐a concentration from ocean colour sensors.	https://oceancolor.gsfc.nasa.gov/	(Bestley et al. 2019; Laidre et al. 2010; Buchan and Quiñones 2016)
Sea Ice Concentration (ICE; km)	NSIDC Sea Ice Index v3	25 km	Monthly	Based on passive microwave data, 15% and 85% thresholds for ice edges.	https://nsidc.org/data/seaice_index	(Nash et al. 2023; Shabangu et al. 2020; Germishuizen et al. 2024)
Sea Surface Temperature (SST; °C)	ODYSSEA Level 4 (MET Norway)	0.1° × 0.1°	Daily	Multi‐sensor SST foundation temperature.	https://doi.org/10.48670/mds‐00321	(Owen et al. 2018; Chambault et al. 2018; Meynecke et al. 2021)
Sea Surface Salinity (SSS; PSU)	CMEMS CNR REP (Level 4)	0.125° × 0.125°	Daily	Derived from SMOS, SMAP, SST and in situ data.	https://doi.org/10.48670/moi‐00051	(Buchan et al. 2022; Buchan and Quiñones 2016)
Sea Surface Height (SSH; m)	CMEMS ARMOR3D REP	0.25° × 0.25°	Weekly	3D geostrophic height from multi‐observation sources.	https://doi.org/10.48670/moi‐00052	(Bombosch et al. 2014; Danielson et al. 2023; Mackay et al. 2020)
Mixed Layer Depth (MLD m)	CMEMS ARMOR3D REP	0.25° × 0.25°	Weekly	Derived from in situ profiles blended with satellite surface data.	https://doi.org/10.48670/moi‐00052	(Johnston et al. 2022; Cassar et al. 2015; Baumgartner and Mate 2003)

*Note:* Each variable was sourced from publicly available remote‐sensing datasets. Citations discussing the ecological significance of each variable, with a focus on the Southern Ocean and baleen whale foraging, are also provided.

Distance to the ice edge was calculated as the great‐circle distance between each whale location and the 15% September ice edge contour. Locations north of the ice edge were assigned positive distances, and locations south of the ice edge were assigned negative distances. This sign distinction was retained because being on either side of the ice margin represents different environmental conditions, and preserving this contrast allows the model to reflect how proximity to, or movement poleward of, the winter ice extent influences predicted foraging suitability. One static covariate, bathymetry (BATHY), was also included (Table 1). These variables were selected based on their established importance in shaping Southern Ocean habitat structure and baleen whale foraging ecology (Reisinger et al. 2021, 2022; Carman et al. 2019).

### 2.8 Temperature—Salinity Diagram

Temperature–salinity (T–S) diagrams were constructed using daily SST and salinity (SSS) values extracted at the whale locations. Points were coloured by behavioural state (area‐restricted search, ARS; directed travel) and plotted within a shared T–S space to allow direct visual comparison of hydrographic conditions occupied during each state. The diagrams were used descriptively to assess point distribution and overlap in relation to broad surface water‐mass characteristics, rather than to perform formal statistical tests of hydrographic differentiation.

### 2.9 Random Forest Model

Random forests were used to predict the probability of ARS behaviour (ARS suitability) in SRWs based on seven environmental covariates. Random forest is a widely used supervised ensemble learning method (Breiman 2001; Fawagreh et al. 2014) and has been extensively applied in ecological studies to model species distributions and habitat suitability (e.g., Reisinger et al. 2021). Models were fitted using the randomForest and caret packages (version 4.7.1.1; Liaw and Wiener 2002; Kuhn et al. 2020). Whale behavioural state was classified using a *γ* = 0.5 threshold derived from the movement persistence model, defining two mutually exclusive states: ARS (*γ* < 0.5) and directed travel (*γ* ≥ 0.5). These binary states were used as the response variable in the random forest classifier. Environmental covariates were extracted at each whale location according to the corresponding observation date across all tracking years (2021–2025), ensuring temporal matching between whale behaviour and environmental conditions.

A single random forest model was fitted using all whale location–environment pairs. The dataset was partitioned at the individual‐track level, with entire tracks assigned exclusively to either the training (70%) or testing (30%) dataset to avoid splitting temporally autocorrelated locations from the same movement trajectory across model partitions. Model parameters were optimised using grid search, with 10‐fold cross‐validation applied to the training set. Model performance was evaluated using the Area Under the Curve (AUC) of the Receiver Operating Characteristic (ROC) curve on the independent test set. Variable importance was assessed using the mean decrease in Gini index, and partial dependence plots were generated to examine relationships between environmental predictors and ARS probability.

To examine the spatial structure of predicted ARS suitability across the broader foraging domain, the fitted random forest model was applied to monthly environmental raster stacks assembled for each month from January 2022 to December 2024. Each stack contained the same seven predictors used during model training. The trained model was applied unchanged to each monthly raster stack to generate monthly suitability maps, representing spatial extrapolations of the relationships between whale behaviour and environmental covariates. Annual mean suitability maps were calculated by averaging monthly predictions within each calendar year for the years with complete tracking coverage (2022–2024).

## 3 Results

**FIGURE 1:**
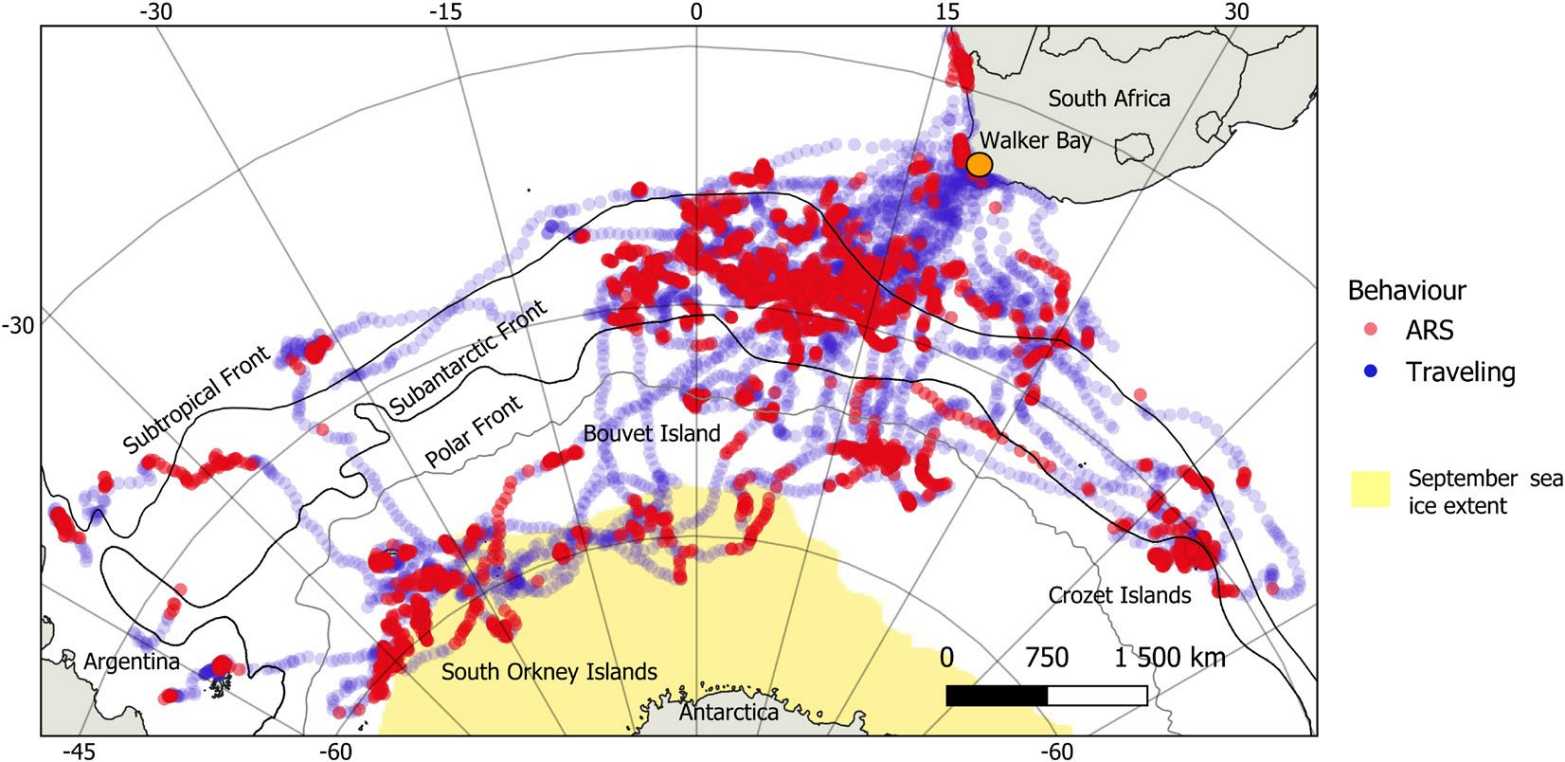
Modelled satellite tracks of southern right whales tagged in Walker Bay, South Africa (large orange dot), in the months of October 2021, 2022, 2023 and 2024 (*n* = 34). Behavioural modes are indicated for each location derived from the continuous movement parameter (*γ*), indicating areas where whales are likely to be foraging (ARS—red) and travelling (blue). ARS is defined as *γ* < 0.5. Lines indicate the mean position of fronts associated with the Antarctic Circumpolar Current (ACC). The yellow shaded region indicates the mean September sea‐ice extent over the tracking period (2021–2024), representing the month wherein the maximum sea‐ice extent is observed.

Whale tracks revealed large spatial variability in movements (Figure 1). Animals generally initiated their migration in a WSW to SE direction before spreading out across the South Atlantic. Areas of high‐use included the mid‐latitudes to the south of South Africa, a high‐latitude region stretching from Bouvet Island to the South Orkney Islands, the Crozet Islands, and the west coast of South Africa (Figure 1). Interestingly, one individual (Tag id: 253548) migrated into the Southern Ocean, reaching ~45° S before returning to feed on the South African west coast, and then returned along a similar track back into the Southern Ocean, with the last transmission at ~39° S (Table S1; Figure S4). Seven individuals undertook transoceanic migrations across the South Atlantic, with several reaching the Patagonian Shelf, off the east coast of South America, and others presumed foraging in high‐latitude waters near South Georgia and the South Sandwich Islands.

**FIGURE 2:**
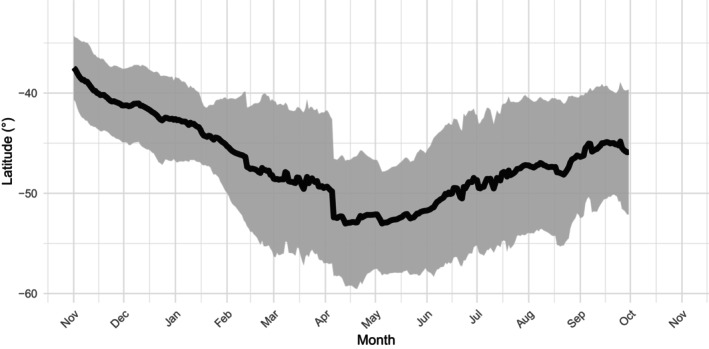
Mean daily latitude over a migratory year (01 November–30 October) pooled across 5 years of tracking. Grey ribbon shows the standard deviation. The migratory year is defined from November to October because tagging occurred in October, creating an artificial latitude jump unrelated to whale movement.

The mean daily latitude for all tagged whales (Figure 2) indicates that they followed a steady southward movement and were typically south of 50° S between April and mid‐June, after which they slowly headed north. A steep decline in latitude is evident from ~48° S to ~54° S during April.

**FIGURE 3:**
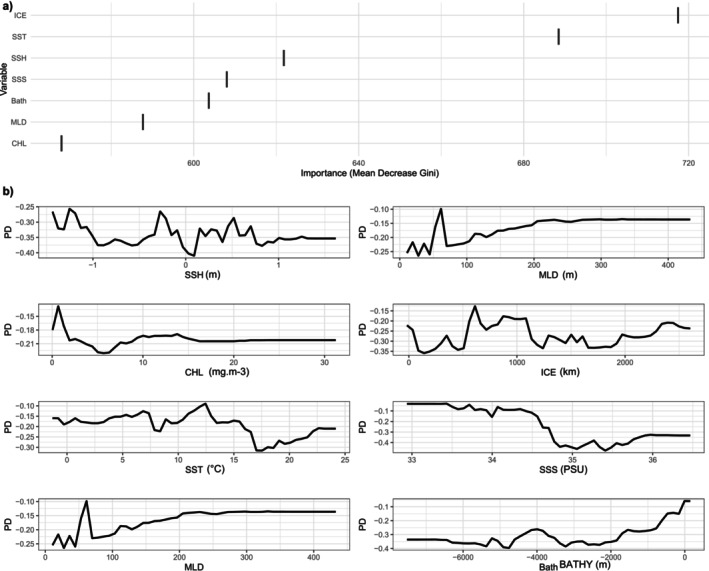
Variable importance and partial dependence plots for the random forest model predicting area‐restricted search (ARS) behaviour in southern right whales. (a) Mean decrease in accuracy for each environmental predictor, indicating relative importance in the random forest model. (b) Partial dependence (PD) plots showing the marginal effect of each variable on ARS probability, holding all other variables constant. Variables include sea surface height (SSH), mixed layer depth (MLD), chlorophyll‐a concentration (CHL), sea ice distance (ICE), sea surface temperature (SST), sea surface salinity (SSS), and bathymetry (BATHY). PD values reflect the model's prediction response across the range of observed environmental values.

A random forest model demonstrated strong predictive performance for identifying ARS behaviour from environmental covariates, with a mean AUC of 0.90 across 10‐fold cross‐validation. Variable importance analysis (Figure 3a) identified ICE and SST as the most influential predictors of ARS behaviour, followed by SSH, SSS, and BATHY. The least important predictors were MLD and CHL. Partial dependence plots (Figure 3b) revealed non‐linear responses of ARS probability to each variable. For example, ARS likelihood increased near steep bathymetric features, with a noticeable peak at approximately −4000 m and increasing likelihood from depths of −2000 to 0 m. SSH revealed a dynamic relationship, with increasing probabilities of ARS at low SSH values (< −1 m), and numerous peaks at intermediate SSH (between −0.5 and 0.5). SST displayed consistent likelihoods of ARS in temperatures < 15°C with multiple peaks likely reflecting the dynamic nature of Southern Ocean fronts. A similar pattern was evident in SSS < 35 psu, demonstrating that feeding occurred in cool, fresher water. MLD exhibited a strong peak in likelihood of ARS at approximately 50 m depth, followed by a gradual increase until 200 m, after which increases in depth had no effect on predicting ARS. Higher probabilities were also observed in waters with intermediate CHL levels, suggesting a preference for persistent but not extreme phytoplankton concentrations. ARS had numerous peaks at various distances from the ICE with a smaller peak observed at the ice edge itself.

**FIGURE 4:**
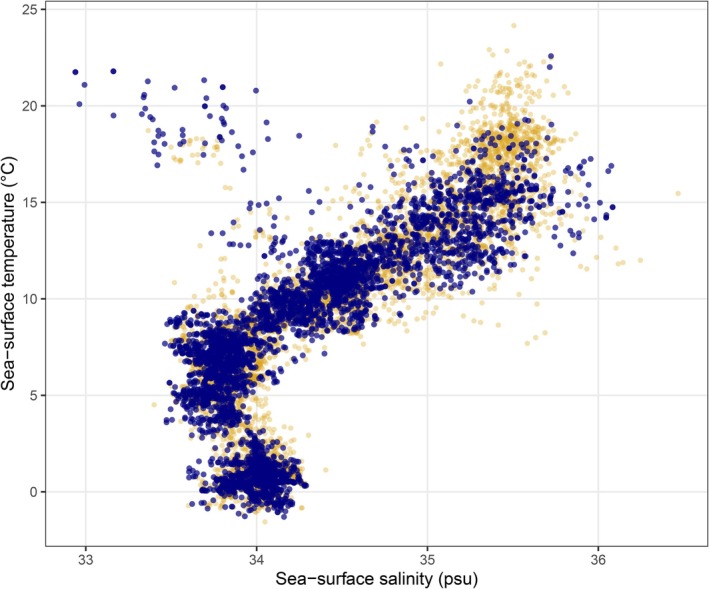
Temperature–salinity (T–S) space occupied by southern right whales during area‐restricted search (ARS; dark blue) and directed travel (gold). Points represent daily sea‐surface temperature (SST) and salinity (SSS) values extracted at whale locations.

T‐S diagrams were used as a visual tool to explore possible differences in hydrographic space occupied during ARS and directed travel (Figure 4). Overall, ARS and travelling locations exhibited a broad and largely overlapping distribution in T‐S space. Points were relatively evenly distributed across the observed hydrographic range, and substantial overlap was evident throughout most of the sampled salinity‐S combinations. Three regions of relatively higher point concentration were apparent. Two clusters occurred within T‐S characteristics typical of Antarctic Circumpolar Current (ACC) waters, broadly corresponding to conditions north and south of the Subantarctic Front. These clusters were not sharply delineated, and ARS locations formed a semi‐continuous band across much of the ACC water‐mass range rather than being confined to discrete T–S signatures. This pattern suggests that ARS behaviour occurs throughout ACC surface waters rather than being associated with a narrowly defined hydrographic class. At warmer (> 13°C) and more saline (> 34.5 PSU) conditions, ARS locations became less frequent, whereas travelling points extended more consistently into these saltier, warmer waters. The clearest visual distinction occurred between the ACC‐associated points and those in the colder, fresher seasonal sea‐ice zone water masses, where ARS locations formed a more distinct grouping at low temperature and moderate salinity. A small, diffuse cluster of ARS points was also visible within hydrographic conditions consistent with low‐salinity Agulhas ring waters, although the limited number of observations precludes firm interpretation.

**FIGURE 5:**
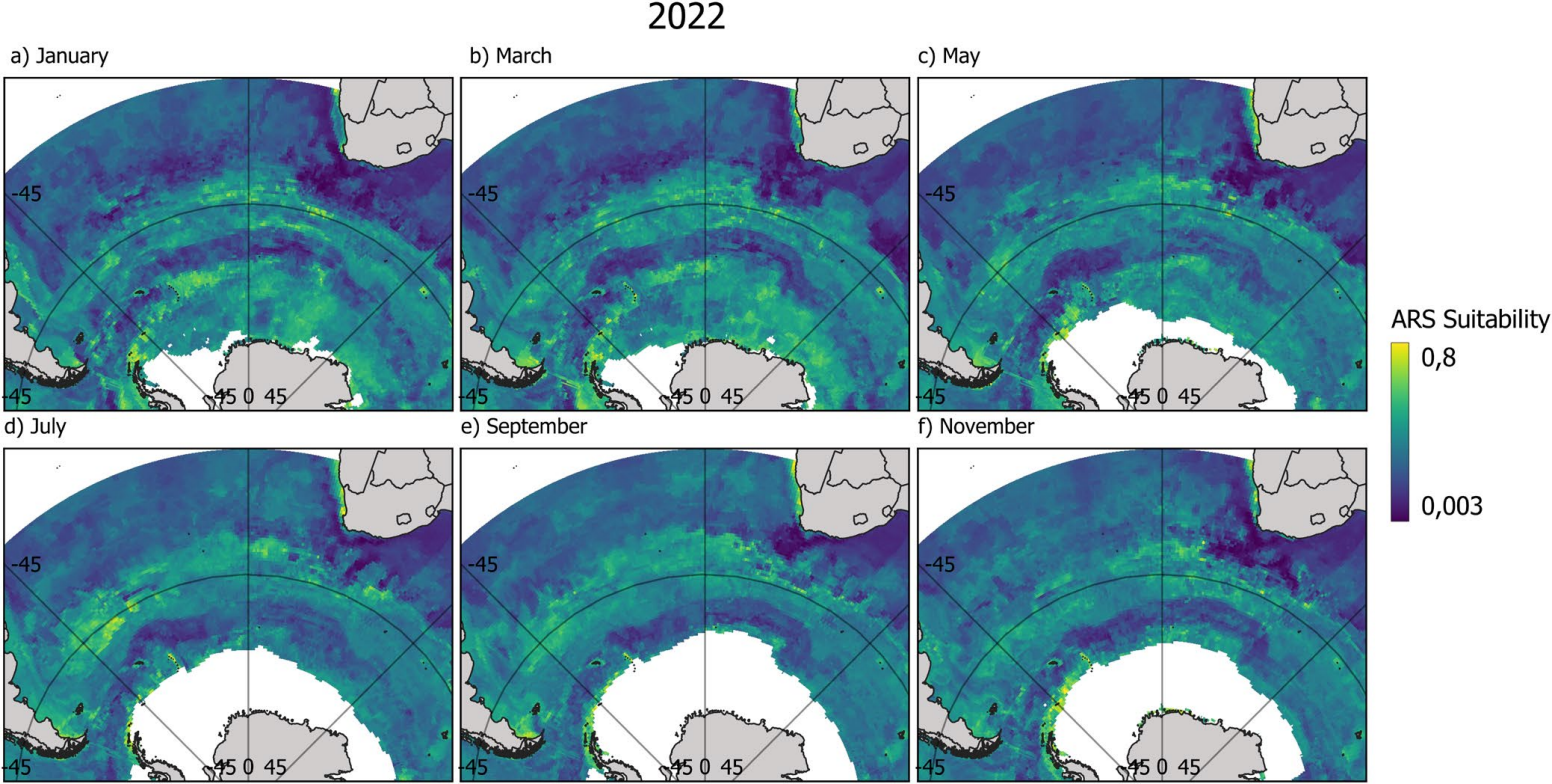
Monthly habitat suitability predictions of area‐restricted search (ARS) behaviour based on random forest models trained using telemetry data from South African southern right whales tagged between 2021 and 2025. Predictions are shown for six representative months: (a) January, (b) March, (c) May, (d) July, (e) September, and (f) November from 2022, a year randomly selected as an illustrative example. Foraging habitat suitability increases from blue to yellow. See Supporting Information for years not presented here. The white region around the Antarctic depicts areas covered by sea ice.

Habitat suitability maps derived from the random forest model were used to assess the spatial distribution and seasonality of favourable ARS habitat. Monthly habitat suitability predictions for 2022 (chosen at random as a representative example; Figure 5) highlighted the dynamic yet spatially structured nature of foraging habitat across the Southern Ocean (see Figures [Link], [Link] for years not presented here). Areas of highest habitat suitability are located along the Antarctic Peninsula, extending to ~0° longitude, and a broadly favourable region in the mid‐latitudes centred at ~ −45° latitude. Furthermore, areas of high suitability are visible on the coastal shelves of western southern Africa and Argentina.

## 4 Discussion

Here, satellite telemetry and habitat modelling provided a contemporary update on the offshore migratory behaviour and foraging ecology of South African SRWs. This is particularly relevant given recent population‐level changes in calving success (Vermeulen et al. 2025) and evidence for shifts in foraging strategy inferred from stable isotope analyses (Derville et al. 2023; van den Berg et al. 2021). The results indicated that the contemporary population predominantly exhibited assumed foraging (ARS) behaviour within mid‐latitude systems of the Southern Ocean, particularly within the ACC and around the Crozet Islands. The seasonal sea‐ice zone was also found to be an important component of habitat use, while productive shelf‐associated systems such as the Benguela upwelling region were also utilised by some individuals. Overall, the findings suggest that habitats associated with assumed foraging behaviour are strongly structured by frontal systems and mesoscale variability within the ACC.

The relationship between the ICE variable and ARS probability indicated that assumed foraging occurred predominantly at intermediate distances from the sea‐ice edge, highlighting the importance of mid‐latitude habitats within the ACC. However, elevated ARS probabilities near the sea‐ice edge also suggest that the marginal ice zone remains an important component of SRW habitat use. SST emerged as the strongest predictor of ARS behaviour, which is unsurprising given the fundamental role of temperature in structuring ocean density gradients, frontal systems, and broader hydrographic organisation across the Southern Ocean. The model suggested that SRW habitat use reflects broad‐scale hydrographic structure, while finer‐scale feeding opportunities are likely associated with mesoscale features such as fronts and eddies. Peaks in the SST partial dependence relationships broadly coincided with the classical definitions of major Southern Ocean frontal systems (Belkin and Gordon 1996; Chapman et al. 2020), suggesting that these fronts may augment feeding opportunities by enhancing prey aggregation and local productivity. Similarly, the strong clustering of ARS behaviour within the eddy‐rich region between South Africa and the Subtropical Front supports growing evidence that mesoscale oceanographic processes play a major role in structuring baleen whale feeding habitat (Carman et al. 2019; Riekkola et al. 2025).

Although the habitat models identified several environmental variables associated with ARS behaviour, the comparatively weak influence of surface chlorophyll and the limited seasonal variability in predicted habitat suitability suggest that important ecological processes may not be fully captured by the environmental predictors used here. Surface chlorophyll is widely used as a proxy for primary productivity, yet it does not resolve subsurface productivity features such as deep chlorophyll maxima, which are increasingly recognised as important components of Southern Ocean ecosystem structure and prey dynamics (Vives et al. 2025). If prey availability is strongly linked to these subsurface features, surface‐based productivity metrics may underestimate the importance of seasonal biological processes in shaping SRW habitat use. Similar patterns have been documented in other baleen whales, including bowhead whales, which target prey associated with subsurface chlorophyll layers (Laidre et al. 2010).

The T–S diagrams were used to investigate broad patterns in water‐mass usage by SRWs. Within the ACC hydrographic domain, ARS and travelling behaviour showed a high degree of overlap in T–S space, indicating that broad water‐mass properties alone do not strongly differentiate ARS from transiting behaviour. Instead, this suggests that habitats associated with ARS behaviour within the ACC are likely structured more strongly by mesoscale variability embedded within broadly suitable hydrographic regions. In contrast, the strongest hydrographic distinction occurred between the ACC and the colder, fresher seasonal sea‐ice zone, which formed a more discrete hydrographic and ecological regime. Together, these patterns suggest that while SRWs continue to utilise both ACC and Antarctic habitats, the relative balance between these systems may be shifting through time.

Strong evidence for shifts in habitat use stems from stable isotope analyses suggesting increasing dietary diversification and northward shifts in feeding areas over recent decades (Derville et al. 2023; van den Berg et al. 2021). The presented telemetry results broadly support this interpretation and, when compared with historical datasets, suggest reduced contemporary use of Antarctic habitats. Historical Soviet whaling records indicate that the Bouvet Island region likely represented a major feeding hotspot for the South African population (Tormosov et al. 1998), a pattern also reflected in the limited 2001/2002 telemetry dataset (Mate et al. 2011). In contrast, contemporary whales displayed reduced use of Antarctic habitats around Bouvet Island and delayed southward movement into high‐latitude waters, with the earliest contemporary crossing of 50° S occurring only in December, whereas most whales in 2001 had crossed this latitude by October (Mate et al. 2011). Together, these patterns suggest a greater contemporary reliance on mid‐latitude habitats, potentially linked to changing sea‐ice conditions and broader shifts in Southern Ocean productivity (Germishuizen et al. 2024).

Despite these apparent shifts, the population still exhibited substantial flexibility in offshore habitat use, utilising the seasonal sea‐ice zone, mid‐latitude frontal systems, and productive shelf‐associated systems such as the Benguela upwelling region. This flexibility likely enables SRWs to exploit a broad range of oceanographic conditions and may partially buffer the population against regional variation in prey availability. Trans‐Atlantic crossings further demonstrate the capacity for basin‐scale migrations and support previous evidence for connectivity among South Atlantic SRW populations (Patenaude et al. 2007; Carroll et al. 2015; Vermeulen, Germishuizen, et al. 2023), while shared habitats associated with ARS behaviour at the Crozet Islands suggest some level of connectivity between South African and Australian populations (Vermeulen et al. 2024). Nevertheless, there is also evidence for maternally directed site fidelity to foraging grounds in southern right whales (Valenzuela et al. 2009), although the spatial scale and rigidity of this fidelity remain poorly understood. Given the substantial variability observed in migratory movements and habitat use among individuals, it is possible that fidelity operates primarily at the scale of broad oceanographic systems and persistent geographic features, such as the ACC, seasonal sea‐ice zone, island systems, and shelf‐associated habitats, while fine‐scale movement and habitat selection within these regions are shaped more dynamically through intrinsic sensory mechanisms involved in prey detection and environmental cueing (Cronin et al. 2017; Farnkopf et al. 2022).

Several limitations associated with the habitat modelling approach should also be acknowledged. The random forest models used here are inherently correlative and therefore cannot demonstrate causal ecological mechanisms linking environmental variability to whale behaviour. Instead, the models identify statistical relationships between behavioural states and environmental conditions. Consequently, interpretations of variable importance and partial dependence relationships should be viewed primarily as ecological associations rather than direct mechanistic responses. This is particularly important given that environmental variables in Southern Ocean ecosystems are strongly interrelated through coupled physical and biological processes (Chapman et al. 2020). Similarly, while distance from the sea‐ice edge emerged as the most important predictor, this variable likely reflects the broad spatial structure of Southern Ocean habitats rather than acting as a direct ecological driver in isolation. In this context, the sea‐ice edge may function more as a large‐scale spatial reference analogous to latitude. Future studies may benefit from incorporating more dynamic descriptors of the seasonal sea‐ice zone, such as sea‐ice concentration, ice‐edge variability, or metrics describing ice retreat timing and persistence.

Interpretation of the hydrographic analyses also remains cautious as tracking data are inherently autocorrelated and individuals contributed unequal numbers of observations. Furthermore, seasonal movements of SRWs are unlikely to be determined solely by short‐term prey availability. Migratory timing and habitat use are also shaped by life‐history constraints, reproductive requirements, and inherited migratory strategies. Because the ACC and seasonal sea‐ice zone are governed by fundamentally different oceanographic processes, a single circumpolar habitat model may also obscure important regional mechanisms. Developing region‐specific habitat models may therefore improve understanding of the environmental drivers underlying seasonal habitat use dynamics.

Overall, these findings substantially advance understanding of the offshore ecology and migratory behaviour of South African SRWs. By integrating telemetry with environmental modelling, the study identifies the principal oceanographic systems associated with ARS behaviour and highlights the importance of mesoscale variability, frontal dynamics, and sea‐ice processes in structuring habitat use across the Southern Ocean. More broadly, the results suggest that SRWs may respond flexibly to environmental variability across multiple spatial scales, enabling the population to exploit a diverse range of productive habitats under changing ocean conditions. Continued monitoring of SRW movements, habitat use, and foraging ecology will therefore remain important for understanding the ecological consequences of rapid environmental change across the Southern Ocean.

## Author Contributions

**Matthew Germishuizen:** conceptualization (equal), formal analysis (lead), investigation (lead), methodology (lead), visualization (lead), writing – original draft (lead), writing – review and editing (lead). **Alexandre N. Zerbini:** conceptualization (equal), data curation (equal), funding acquisition (lead), methodology (equal), project administration (equal), software (equal), writing – original draft (supporting), writing – review and editing (equal). **Amy Kennedy:** data curation (equal), writing – review and editing (equal). **Marcello Vichi:** conceptualization (equal), formal analysis (equal), writing – review and editing (equal). **Christopher Wilkinson:** data curation (equal), writing – review and editing (equal). **Els Vermeulen:** conceptualization (equal), data curation (lead), funding acquisition (lead), project administration (lead), resources (equal), supervision (lead), writing – review and editing (equal).

## Funding

This work was supported by Cooperative Institute for Climate, Ocean & Ecosystem Studies (CICOES), 2025‐1510, South African Polar Research Infrastructure, WWF Protecting Whales & Dolphins Initiative, Marine Ecology and Telemetry Research (MarEcoTel) and Instituto Aqualie.

## Conflicts of Interest

The authors declare no conflicts of interest.

## Supporting information


**Figure S1:** Individual movement tracks of southern right whales ( *Eubalaena australis* ) deployed in Walker Bay, South Africa in 2021. Each panel shows the track of a tagged whale, faceted by ID.

**Figure S2:** Individual movement tracks of southern right whales ( *Eubalaena australis* ) deployed in Walker Bay, South Africa in 2022. Each panel shows the track of a tagged whale, faceted by ID.

**Figure S3:** Individual movement tracks of southern right whales ( *Eubalaena australis* ) deployed in Walker Bay, South Africa in 2023. Each panel shows the track of a tagged whale, faceted by ID.

**Figure S4:** Individual movement tracks of southern right whales ( *Eubalaena australis* ) deployed in Walker Bay, South Africa in 2024. Each panel shows the track of a tagged whale, faceted by ID.

**Figure S5:** Monthly habitat suitability predictions of area‐restricted search (ARS) behaviour based on random forest models trained using telemetry data from South African southern right whales tagged between 2021 and 2025. Predictions are shown for six representative months: (a) February, (b) April, (c) June, (d) August, (e) October, and (f) December from 2022. Foraging habitat suitability increases from blue to yellow.

**Figure S6:** Monthly habitat suitability predictions of area‐restricted search (ARS) behaviour based on random forest models trained using telemetry data from South African southern right whales tagged between 2021 and 2025. Predictions are shown for six representative months: (a) January, (b) March, (c) May, (d) July, (e) September, and (f) November from 2023. Foraging habitat suitability increases from blue to yellow.

**Figure S7:** Monthly habitat suitability predictions of area‐restricted search (ARS) behaviour based on random forest models trained using telemetry data from South African southern right whales tagged between 2021 and 2025. Predictions are shown for six representative months: (a) February, (b) April, (c) June, (d) August, (e) October, and (f) December from 2023. Foraging habitat suitability increases from blue to yellow.

**Figure S8:** Monthly habitat suitability predictions of area‐restricted search (ARS) behaviour based on random forest models trained using telemetry data from South African southern right whales tagged between 2021 and 2025. Predictions are shown for six representative months: (a) January, (b) March, (c) May, (d) July, (e) September, and (f) November from 2024. Foraging habitat suitability increases from blue to yellow.

**Figure S9:** Monthly habitat suitability predictions of area‐restricted search (ARS) behaviour based on random forest models trained using telemetry data from South African southern right whales tagged between 2021 and 2025. Predictions are shown for six representative months: (a) February, (b) April, (c) June, (d) August, (e) October, and (f) December from 2024. Foraging habitat suitability increases from blue to yellow.

**Figure S10:** Locations of satellite‐tracked Southern right whale coloured according to the estimated movement persistence parameter (*γ*) derived from the movement model. Lower values (purple/blue) indicate more tortuous movement associated with area‐restricted search or potential foraging behaviour, whereas higher values (green/yellow) indicate more directed movement consistent with transiting or migratory behaviour.

**Table S1:** Summary of the 34 satellite‐tracked southern right whale movements. Tags that were still transmitting at the time of paper writing are denoted with an Asterix (*). For animals that were genetically sexed (see Methods), the sex is shown in brackets after the Tag ID (M or F).

## Data Availability

Environmental data, whale locations and the random forest model used in the study are available here: https://doi.org/10.5281/zenodo.17864770. All environmental variables were obtained from publicly accessible online repositories, as detailed in the Methods section.

## References

[ece373975-bib-0001] Agrelo, M. , F. G. Daura‐Jorge , V. J. Rowntree , et al. 2021. “Ocean Warming Threatens Southern Right Whale Population Recovery.” Science Advances 7, no. 42: eabh2823. 10.1126/sciadv.abh2823. 34652948 PMC8519561

[ece373975-bib-0002] Baumgartner, M. F. , and B. R. Mate . 2003. “Summertime Foraging Ecology of North Atlantic Right Whales.” Marine Ecology Progress Series 264: 123–135.

[ece373975-bib-0003] Belkin, I. M. , and A. L. Gordon . 1996. “Southern Ocean Fronts From the Greenwich Meridian to Tasmania.” Journal of Geophysical Research: Oceans 101, no. C2: 3675–3696.

[ece373975-bib-0004] Bestley, S. , V. Andrews‐Goff , E. van Wijk , S. R. Rintoul , M. C. Double , and J. How . 2019. “New Insights Into Prime Southern Ocean Forage Grounds for Thriving Western Australian Humpback Whales.” Scientific Reports 9, no. 1: 13988. 31562374 10.1038/s41598-019-50497-2 PMC6764985

[ece373975-bib-0005] Bombosch, A. , D. P. Zitterbart , I. Van Opzeeland , et al. 2014. “Predictive Habitat Modelling of Humpback ( *Megaptera novaeangliae* ) and Antarctic Minke ( *Balaenoptera bonaerensis* ) Whales in the Southern Ocean as a Planning Tool for Seismic Surveys.” Deep Sea Research Part I: Oceanographic Research Papers 91: 101–114.

[ece373975-bib-0006] Breiman, L. 2001. “Random Forests.” Machine Learning 45, no. 1: 5–32.

[ece373975-bib-0007] Buchan, S. J. , L. Gutiérrez , M. F. Baumgartner , et al. 2022. “Distribution of Blue and Sei Whale Vocalizations, and Temperature‐Salinity Characteristics From Glider Surveys in the Northern Chilean Patagonia Mega‐Estuarine System.” Frontiers in Marine Science 9: p.903964.

[ece373975-bib-0008] Buchan, S. J. , and R. A. Quiñones . 2016. “First Insights Into the Oceanographic Characteristics of a Blue Whale Feeding Ground in Northern Patagonia, Chile.” Marine Ecology Progress Series 554: 183–199.

[ece373975-bib-0009] Carman, V. G. , A. Piola , T. D. O'Brien , D. D. Tormosov , and E. M. Acha . 2019. “Circumpolar Frontal Systems as Potential Feeding Grounds of Southern Right Whales.” Progress in Oceanography 176: 102123.

[ece373975-bib-0010] Carroll, E. , N. Patenaude , A. Alexander , et al. 2011. “Population Structure and Individual Movement of Southern Right Whales Around New Zealand and Australia.” Marine Ecology Progress Series 432: 257–268. 10.3354/meps09145.

[ece373975-bib-0011] Carroll, E. L. , C. S. Baker , M. Watson , et al. 2015. “Cultural Traditions Across a Migratory Network Shape the Genetic Structure of Southern Right Whales Around Australia and New Zealand.” Scientific Reports 5, no. 1: p.16182. 10.1038/srep16182 PMC4637828 26548756

[ece373975-bib-0012] Cassar, N. , S. W. Wright , P. G. Thomson , et al. 2015. “The Relation of Mixed‐Layer Net Community Production to Phytoplankton Community Composition in the Southern Ocean.” Global Biogeochemical Cycles 29, no. 4: 446–462.

[ece373975-bib-0013] Chambault, P. , C. M. Albertsen , T. A. Patterson , et al. 2018. “Sea Surface Temperature Predicts the Movements of an Arctic Cetacean: The Bowhead Whale.” Scientific Reports 8, no. 1: 9658. 29942009 10.1038/s41598-018-27966-1 PMC6018504

[ece373975-bib-0014] Chapman, C. C. , M. A. Lea , A. Meyer , J. B. Sallée , and M. Hindell . 2020. “Defining Southern Ocean Fronts and Their Influence on Biological and Physical Processes in a Changing Climate.” Nature Climate Change 10, no. 3: 209–219.

[ece373975-bib-0015] Charlton, C. , M. Germishuizen , B. O'Shannessy , et al. 2026. “Climate‐Driven Reproductive Decline in Southern Right Whales.” Scientific Reports 16: 5352. 10.1038/s41598-026-36897-1. 41673062 PMC12894980

[ece373975-bib-0016] Crespo, E. A. , S. N. Pedraza , S. L. Dans , G. M. Svendsen , M. Degrati , and M. A. Coscarella . 2019. “The Southwestern Atlantic Southern Right Whale, *Eubalaena australis* , Population Is Growing but at a Decelerated Rate.” Marine Mammal Science 35, no. 1: 93–107. 10.1111/mms.12526.

[ece373975-bib-0017] Cronin, T. W. , J. I. Fasick , L. E. Schweikert , S. Johnsen , L. J. Kezmoh , and M. F. Baumgartner . 2017. “Coping With Copepods: Do Right Whales ( *Eubalaena glacialis* ) Forage Visually in Dark Waters?” Philosophical Transactions of the Royal Society, B: Biological Sciences 372, no. 1717: 20160067. 10.1098/rstb.2016.0067 PMC5312017 28193812

[ece373975-bib-0018] Danielson, R. E. , H. Shen , J. Tao , and W. Perrie . 2023. “Dependence of Ocean Surface Filaments on Wind Speed: An Observational Study of North Atlantic Right Whale Habitat.” Remote Sensing of Environment 287: 113494.

[ece373975-bib-0019] Derville, S. , L. G. Torres , S. D. Newsome , et al. 2023. “Long‐Term Stability in the Circumpolar Foraging Range of a Southern Ocean Predator Between the Eras of Whaling and Rapid Climate Change.” Proceedings of the National Academy of Sciences of the United States of America 120, no. 10: e2214035120. 10.1073/pnas.2214035120. 36848574 PMC10013836

[ece373975-bib-0020] Farnkopf, I. C. , J. C. George , T. Kishida , D. J. Hillmann , R. S. Suydam , and J. G. M. Thewissen . 2022. “Olfactory Epithelium and Ontogeny of the Nasal Chambers in the Bowhead Whale ( *Balaena mysticetus* ).” Anatomical Record 305, no. 3: 643–667. 10.1002/ar.24682 34117725

[ece373975-bib-0021] Fawagreh, K. , M. M. Gaber , and E. Elyan . 2014. “Random Forests: From Early Developments to Recent Advancements.” Systems Science and Control Engineering 2, no. 1: 602–609.

[ece373975-bib-0022] Florko, K. R. , C. R. Shuert , W. W. Cheung , et al. 2023. “Linking Movement and Dive Data to Prey Distribution Models: New Insights in Foraging Behaviour and Potential Pitfalls of Movement Analyses.” Movement Ecology 11, no. 1: 17. 36959671 10.1186/s40462-023-00377-2 PMC10037791

[ece373975-bib-0066] Freitas, C. , K. M. Kovacs , R. A. Ims , and C. Lydersen , 2008. “Predicting Habitat use by Ringed Seals (Phoca hispida) in a Warming Arctic.” Ecological Modelling 217, no. 1–2: 19–32. 10.1016/j.ecolmodel.2008.05.014.

[ece373975-bib-0023] Garrigue, C. , P. J. Clapham , Y. Geyer , A. S. Kennedy , and A. N. Zerbini . 2015. “Satellite Tracking Reveals Novel Migratory Patterns and the Importance of Seamounts for Endangered South Pacific Humpback Whales.” Royal Society Open Science 2, no. 11: 150489. 26716006 10.1098/rsos.150489 PMC4680621

[ece373975-bib-0024] Germishuizen, M. , M. Vichi , and E. Vermeulen . 2024. “Population Changes in a Southern Ocean Krill Predator Point Towards Regional Antarctic Sea Ice Declines.” Scientific Reports 14, no. 1: 25820. 10.1038/s41598-024-74007-1. 39468232 PMC11519949

[ece373975-bib-0025] Grundlehner, A. , J. N. Smith , J. L. Bannister , et al. 2025. “The End of an Era? Trends in Abundance and Reproduction of Australian Southern Right Whales ( *Eubalaena australis* ) Suggest Failure to re‐Establish Pre‐Whaling Population Size.” Global Change Biology 31, no. 5: e70218. 40304046 10.1111/gcb.70218 PMC12042069

[ece373975-bib-0026] Harrison, L. M. K. , K. Goetz , M. J. Cox , and R. Harcourt . 2020. “A Southern Ocean Archipelago Enhances Feeding Opportunities for a Krill Predator.” Marine Mammal Science 36, no. 1: 260–275.

[ece373975-bib-0027] Heide‐Jørgensen, M. P. , L. Kleivane , N. Øien , K. L. Laidre , and M. V. Jensen . 2001. “A New Technique for Deploying Satellite Transmitters on Baleen Whales: Tracking a Blue Whale ( *Balaenoptera musculus* ) in the North Atlantic.” Marine Mammal Science 17, no. 4: 949–954. 10.1111/j.1748-7692.2001.tb01309.x.

[ece373975-bib-0028] Ichii, T. , H. Igarashi , M. Mori , K. Mahapatra , H. Nishikawa , and T. Okuda . 2023. “Impact of the Climate Regime Shift Around 2000 on Recruitment of Antarctic Krill at the Antarctic Peninsula and South Georgia.” Progress in Oceanography 213: 103020.

[ece373975-bib-0029] Johnston, N. M. , E. J. Murphy , A. Atkinson , et al. 2022. “Status, Change, and Futures of Zooplankton in the Southern Ocean.” Frontiers in Ecology and Evolution 9: 624692.

[ece373975-bib-0030] Jonsen, I. D. , W. J. Grecian , L. Phillips , et al. 2023. “aniMotum, an R Package for Animal Movement Data: Rapid Quality Control, Behavioural Estimation and Simulation.” Methods in Ecology and Evolution 14, no. 3: 806–816. 10.1111/2041-210X.14060.

[ece373975-bib-0031] Kennedy, A. S. , E. L. Carroll , A. N. Zerbini , et al. 2023. “Photo‐Identification and Satellite Telemetry Connect Southern Right Whales From South Georgia Island (Islas Georgias Del Sur) With Multiple Feeding and Calving Grounds in the Southwest Atlantic.” Marine Mammal Science 40, no. 2: e13089. 10.1111/mms.13089.

[ece373975-bib-0032] Kuhn, M. , J. Wing , S. Weston , et al. 2020. “Package ‘Caret’.” R Journal 223, no. 7: 48.

[ece373975-bib-0033] Laidre, K. L. , M. P. Heide‐Jørgensen , M. L. Logsdon , L. Delwiche , and T. G. Nielsen . 2010. “A Whale of an Opportunity: Examining the Vertical Structure of Chlorophyll‐a in High Arctic Waters Using Instrumented Marine Predators.” Marine Biology Research 6, no. 6: 519–529.

[ece373975-bib-0034] Liaw, A. , and M. Wiener . 2002. “Classification and Regression by randomForest.” R News 2, no. 3: 18–22.

[ece373975-bib-0035] Lydersen, C. , J. Vacquié‐Garcia , M. P. Heide‐Jørgensen , N. Øien , C. Guinet , and K. M. Kovacs . 2020. “Autumn Movements of Fin Whales ( *Balaenoptera physalus* ) From Svalbard, Norway, Revealed by Satellite Tracking.” Scientific Reports 10, no. 1: 16966. 33046805 10.1038/s41598-020-73996-z PMC7550606

[ece373975-bib-0036] Mackay, A. I. , F. Bailleul , E. L. Carroll , et al. 2020. “Satellite Derived Offshore Migratory Movements of Southern Right Whales ( *Eubalaena australis* ) From Australian and New Zealand Wintering Grounds.” PLoS One 15, no. 5: e0231577. 10.1371/JOURNAL.PONE.0231577. 32380516 PMC7205476

[ece373975-bib-0037] Mate, B. R. , P. B. Best , B. A. Lagerquist , and M. H. Winsor . 2011. “Coastal, Offshore and Migratory Movements of South African Right Whales Revealed by Satellite Telemetry.” Marine Mammal Science 27, no. 3: 455–476.

[ece373975-bib-0038] Meynecke, J. O. , J. De Bie , J. L. M. Barraqueta , et al. 2021. “The Role of Environmental Drivers in Humpback Whale Distribution, Movement and Behavior: A Review.” Frontiers in Marine Science 8: 720774.

[ece373975-bib-0039] Nash, S. M. B. , J. Groβ , J. Castrillon , et al. 2023. “Antarctic Sea‐Ice Low Resonates in the Ecophysiology of Humpback Whales.” Science of the Total Environment 887: 164053. 37178847 10.1016/j.scitotenv.2023.164053

[ece373975-bib-0040] O'Shannessy, B. , L. Möller , R. D. McCauley , et al. 2025. “Decadal Shifts in Southern Right Whale ( *Eubalaena australis* ) Recovery in South Australian Waters: Implications for Conservation and Management.” Marine Mammal Science 41, no. 4: e70045.

[ece373975-bib-0041] Owen, K. , K. C. S. Jenner , M. N. M. Jenner , R. D. McCauley , and R. D. Andrews . 2018. “Water Temperature Correlates With Baleen Whale Foraging Behaviour at Multiple Scales in the Antarctic.” Marine and Freshwater Research 70, no. 1: 19–32.

[ece373975-bib-0042] Patenaude, N. J. , V. A. Portway , C. M. Schaeff , et al. 2007. “Mitochondrial DNA Diversity and Population Structure Among Southern Right Whales ( *Eubalaena australis* ).” Journal of Heredity 98, no. 2: 147–157. 10.1093/jhered/esm005. 17416933

[ece373975-bib-0043] R Core Team . 2024. R: A Language and Environment for Statistical Computing. R Foundation for Statistical Computing. https://www.R‐project.org/.

[ece373975-bib-0044] Reisinger, R. R. , C. M. Brooks , B. Raymond , et al. 2022. “Predator‐Derived Bioregions in the Southern Ocean: Characteristics, Drivers and Representation in Marine Protected Areas.” Biological Conservation 272: 109630.

[ece373975-bib-0045] Reisinger, R. R. , A. S. Friedlaender , A. N. Zerbini , et al. 2021. “Combining Regional Habitat Selection Models for Large‐Scale Prediction: Circumpolar Habitat Selection of Southern Ocean Humpback Whales.” Remote Sensing 13, no. 11: 2074. 10.3390/rs13112074.

[ece373975-bib-0046] Riekkola, L. , K. R. Sprogis , A. Della Penna , et al. 2025. “Large‐Scale Differences, Mesoscale Similarities: Neighbouring Marine Predator Populations Provide Insights Into Southern Ocean Productivity.” Global Ecology and Conservation 62: e03788.

[ece373975-bib-0048] Schroeter, S. , T. J. O'Kane , and P. A. Sandery . 2023. “Antarctic Sea Ice Regime Shift Associated With Decreasing Zonal Symmetry in the Southern Annular Mode.” Cryosphere 17, no. 2: 701–717.

[ece373975-bib-0049] Seyboth, E. , K. R. Groch , L. Dalla Rosa , K. Reid , P. A. C. Flores , and E. R. Secchi . 2016. “Southern Right Whale ( *Eubalaena australis* ) Reproductive Success Is Influenced by Krill ( *Euphausia superba* ) Density and Climate.” Scientific Reports 6: 28205. 10.1038/srep28205. 27306583 PMC4910057

[ece373975-bib-0050] Shabangu, F. W. , R. K. Andrew , D. Yemane , and K. P. Findlay . 2020. “Acoustic Seasonality, Behaviour and Detection Ranges of Antarctic Blue and Fin Whales Under Different Sea Ice Conditions Off Antarctica.” Endangered Species Research 43: 21–37.

[ece373975-bib-0051] Shuert, C. R. , N. E. Hussey , M. Marcoux , M. P. Heide‐Jørgensen , R. Dietz , and M. Auger‐Méthé . 2023. “Divergent Migration Routes Reveal Contrasting Energy‐Minimization Strategies to Deal With Differing Resource Predictability.” Movement Ecology 11, no. 1: 31. 37280701 10.1186/s40462-023-00397-y PMC10245675

[ece373975-bib-0052] Thums, M. , L. C. Ferreira , C. Jenner , et al. 2022. “Pygmy Blue Whale Movement, Distribution and Important Areas in the Eastern Indian Ocean.” Global Ecology and Conservation 35: e02054.

[ece373975-bib-0053] Tormosov, D. D. , Y. A. Mikhaliev , P. B. Best , V. A. Zemsky , K. Sekiguchi , and R. L. Brownell . 1998. “Soviet Catches of Southern Right Whales Eubalaena Australis, 1951–1971. Biological Data and Conservation Implications.” Biological Conservation 86: 185–197. 10.1016/S0006-3207(98)00008-1.

[ece373975-bib-0054] Tulloch, V. J. , É. E. Plagányi , R. Matear , C. J. Brown , and A. J. Richardson . 2018. “Ecosystem Modelling to Quantify the Impact of Historical Whaling on Southern Hemisphere Baleen Whales.” Fish and Fisheries 19, no. 1: 117–137.

[ece373975-bib-0055] Valenzuela, L. O. , M. Sironi , V. J. Rowntree , and J. Seger . 2009. “Isotopic and Genetic Evidence for Culturally Inherited Site Fidelity to Feeding Grounds in Southern Right Whales ( *Eubalaena australis* ).” Molecular Ecology 18, no. 5: 782–791. 19207250 10.1111/j.1365-294X.2008.04069.x

[ece373975-bib-0056] van den Berg, G. L. , E. Vermeulen , L. O. Valenzuela , et al. 2021. “Decadal Shift in Foraging Strategy of a Migratory Southern Ocean Predator.” Global Change Biology 27: 1052–1067. 10.1111/gcb.15465. 33319502

[ece373975-bib-0057] Vermeulen, E. , M. Germishuizen , A. Kennedy , C. Wilkinson , C. R. Weir , and A. Zerbini . 2023. “Swimming Across the Pond: First Documented Transatlantic Crossing of a Southern Right Whale.” Marine Mammal Science 40: 309–316. 10.1111/mms.13071.

[ece373975-bib-0058] Vermeulen, E. , T. Thavar , M. Glarou , A. Ganswindt , and F. Christiansen . 2023. “Decadal Decline in Maternal Body Condition of a Southern Ocean Capital Breeder.” Scientific Reports 13, no. 1: 3228. 10.1038/s41598-023-30238-2. 36828886 PMC9958138

[ece373975-bib-0059] Vermeulen, E. , P. Tixier , E. L. Carroll , et al. 2024. “Multi‐Method Observations Suggest Recolonization of the Crozet Islands by Southern Right Whales With Links to Different Coastal Calving Grounds.” Presented to the International Whaling Commission Scientific Committee Meeting.

[ece373975-bib-0060] Vermeulen, E. , C. Wilkinson , P. B. Best , and K. Findlay . 2025. “Four Decades of Annual Monitoring Reveal Declining Reproductive Success of a Migratory Baleen Whale.” Scientific Reports 15, no. 1: 34713. 41053107 10.1038/s41598-025-18252-y PMC12500924

[ece373975-bib-0061] Vives, C. R. , C. Schallenberg , P. G. Strutton , J. Bendtsen , K. Richardson , and P. W. Boyd . 2025. “The Contribution of Deep Chlorophyll Maxima to Net Primary Production in the Southern Ocean.” Global Biogeochemical Cycles 39, no. 10: p.e2024GB008327.

[ece373975-bib-0062] Watson, M. , K. Stamation , C. Charlton , and J. Bannister . 2021. “Calving Intervals, Longrange Movements and Site Fidelity of Southern Right Whales ( *Eubalaena australis* ) in Southeastern Australia.” Journal of Cetacean Research and Management 22, no. 1: 17–28. 10.47536/JCRM.V22I1.210.

[ece373975-bib-0063] Weir, C. R. , S. Fernandez , J. A. Jackson , et al. 2024. “Movements and Behaviour of Southern Right Whales Satellite‐Tracked in and Beyond a Subantarctic Archipelago Wintering Ground.” Endangered Species Research 55: 229–245.

[ece373975-bib-0064] Zerbini, A. N. , A. Andriolo , M. P. Heide‐Jørgensen , et al. 2006. “Satellite‐Monitored Movements of Humpback Whales *Megaptera novaeangliae* in the Southwest Atlantic Ocean.” Marine Ecology Progress Series 313: 295–304.

[ece373975-bib-0065] Zerbini, A. N. , J. Robbins , V. Andrews‐Goff , et al. 2025. “Developing Robust Large Whale Satellite Tags Through Follow‐Up Studies.” Journal of Cetacean Research and Management Special Issue 5: 91–126. 10.47536/jcrm.v5i1.1091.

