# Supplementary figures and images for: Dynamic Migratory Strategies and Foraging Habitats of Southern Right Whales Revealed by Satellite Telemetry
Source: Ecol Evol. 2026 Jul 6;16(7):e73975. doi: 10.1002/ece3.73975 (PMC13337319; doi:10.1002/ece3.73975)

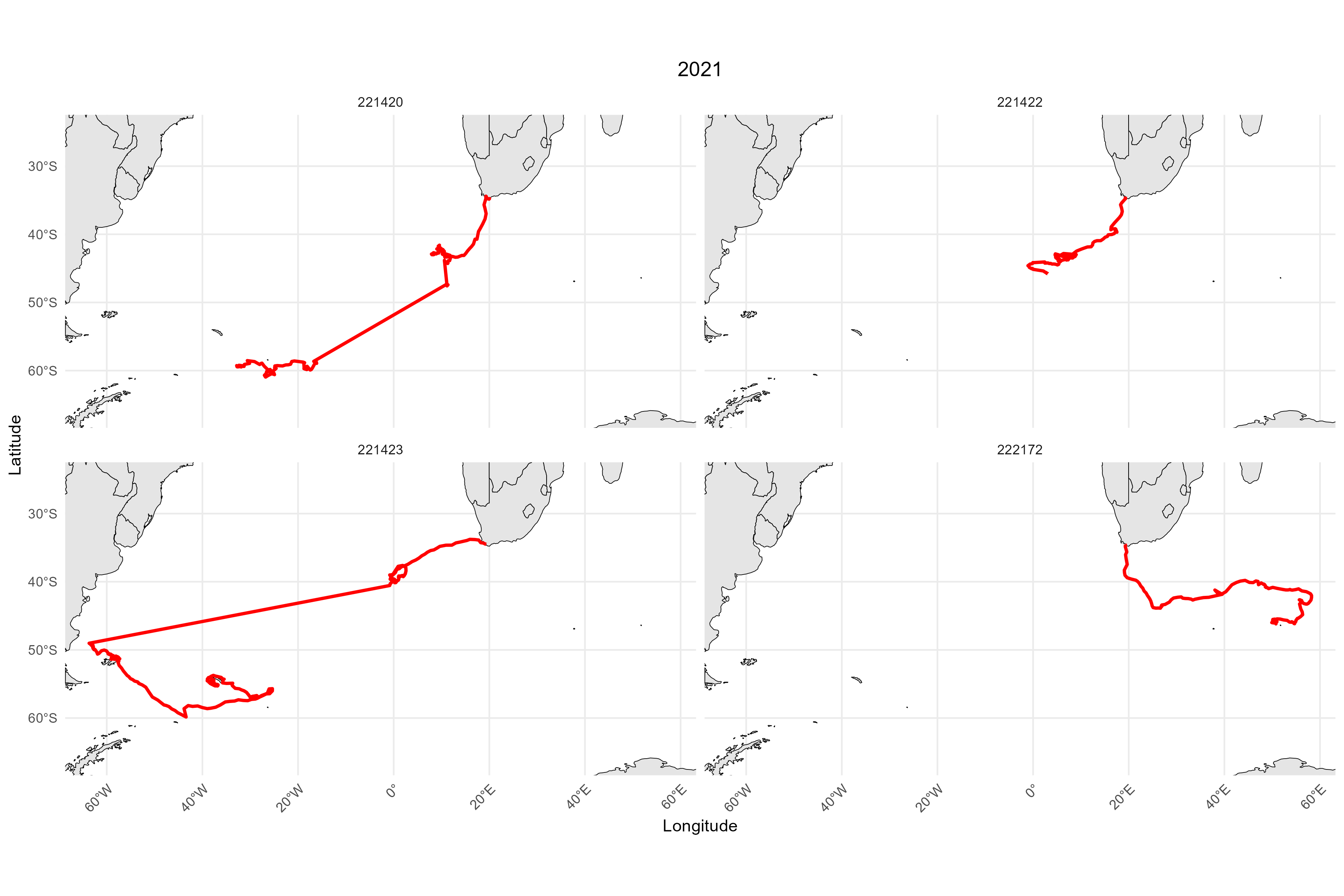

Supplement: Supplementary file 1 — Figure S1: Individual movement tracks of southern right whales ( Eubalaena australis ) deployed in Walker Bay, South Africa in 2021. Each panel shows the track of a tagged whale, faceted by ID. [file ECE3-16-e73975-s002.png]

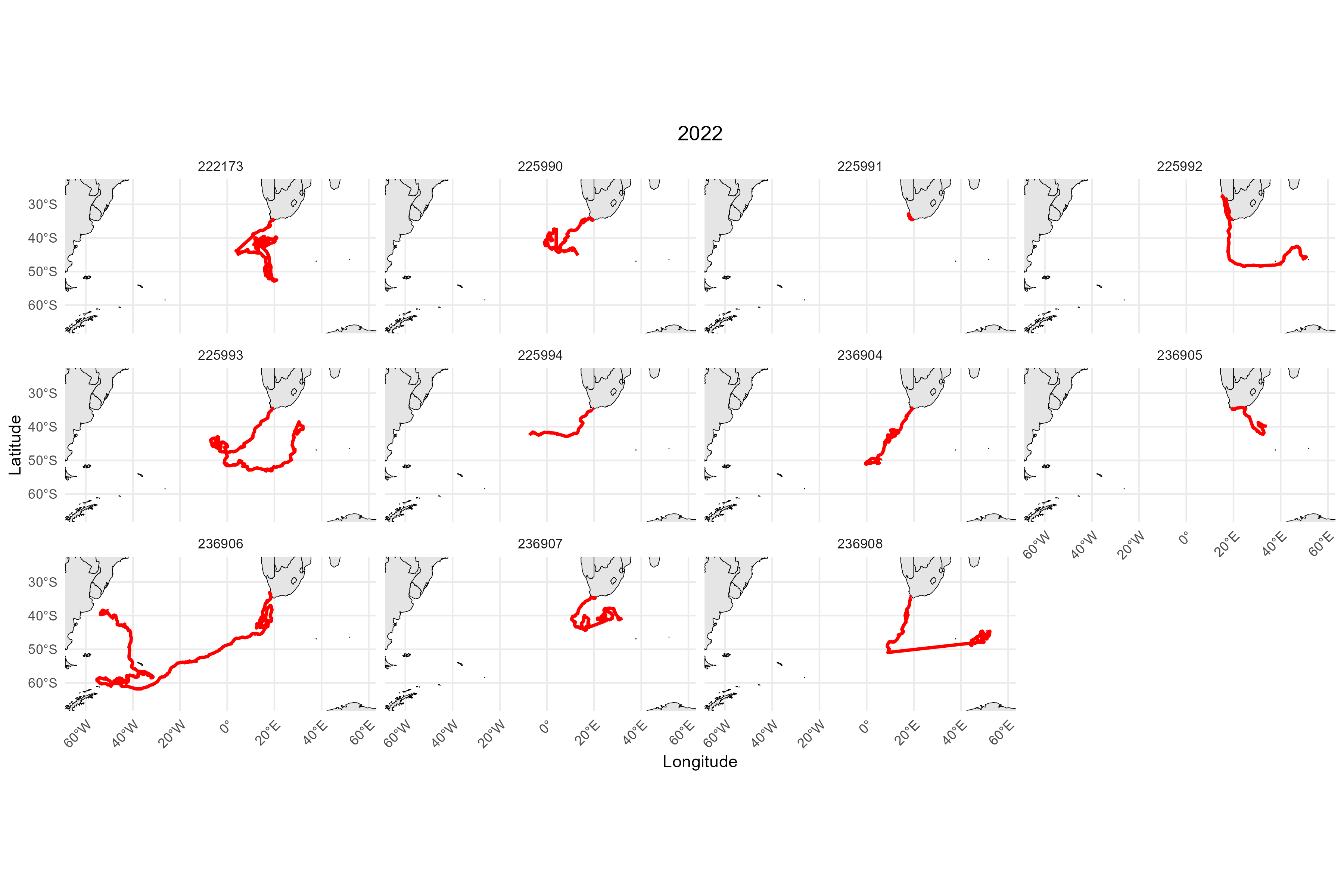

Supplement: Supplementary file 2 — Figure S2: Individual movement tracks of southern right whales ( Eubalaena australis ) deployed in Walker Bay, South Africa in 2022. Each panel shows the track of a tagged whale, faceted by ID. [file ECE3-16-e73975-s004.png]

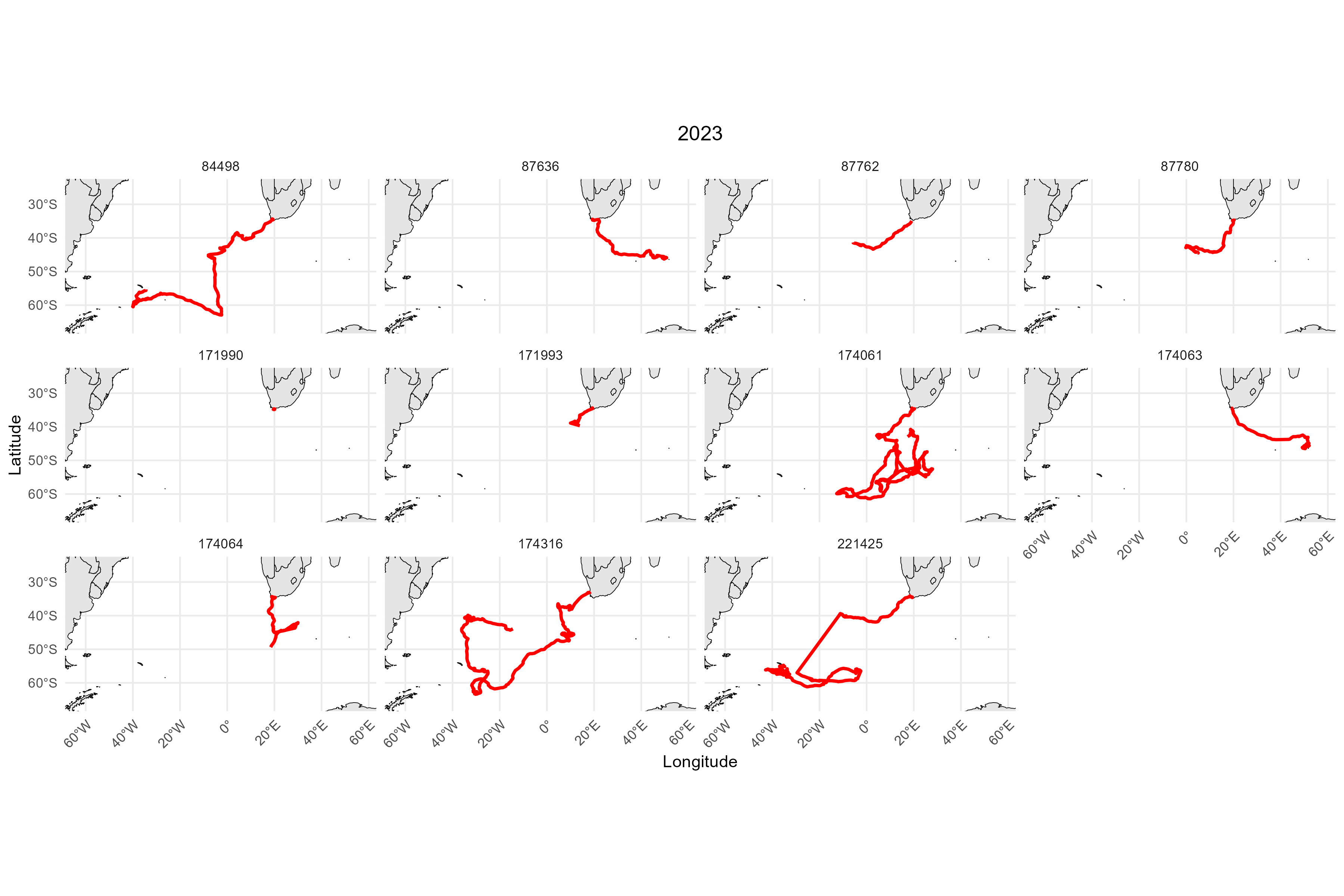

Supplement: Supplementary file 3 — Figure S3: Individual movement tracks of southern right whales ( Eubalaena australis ) deployed in Walker Bay, South Africa in 2023. Each panel shows the track of a tagged whale, faceted by ID. [file ECE3-16-e73975-s010.png]

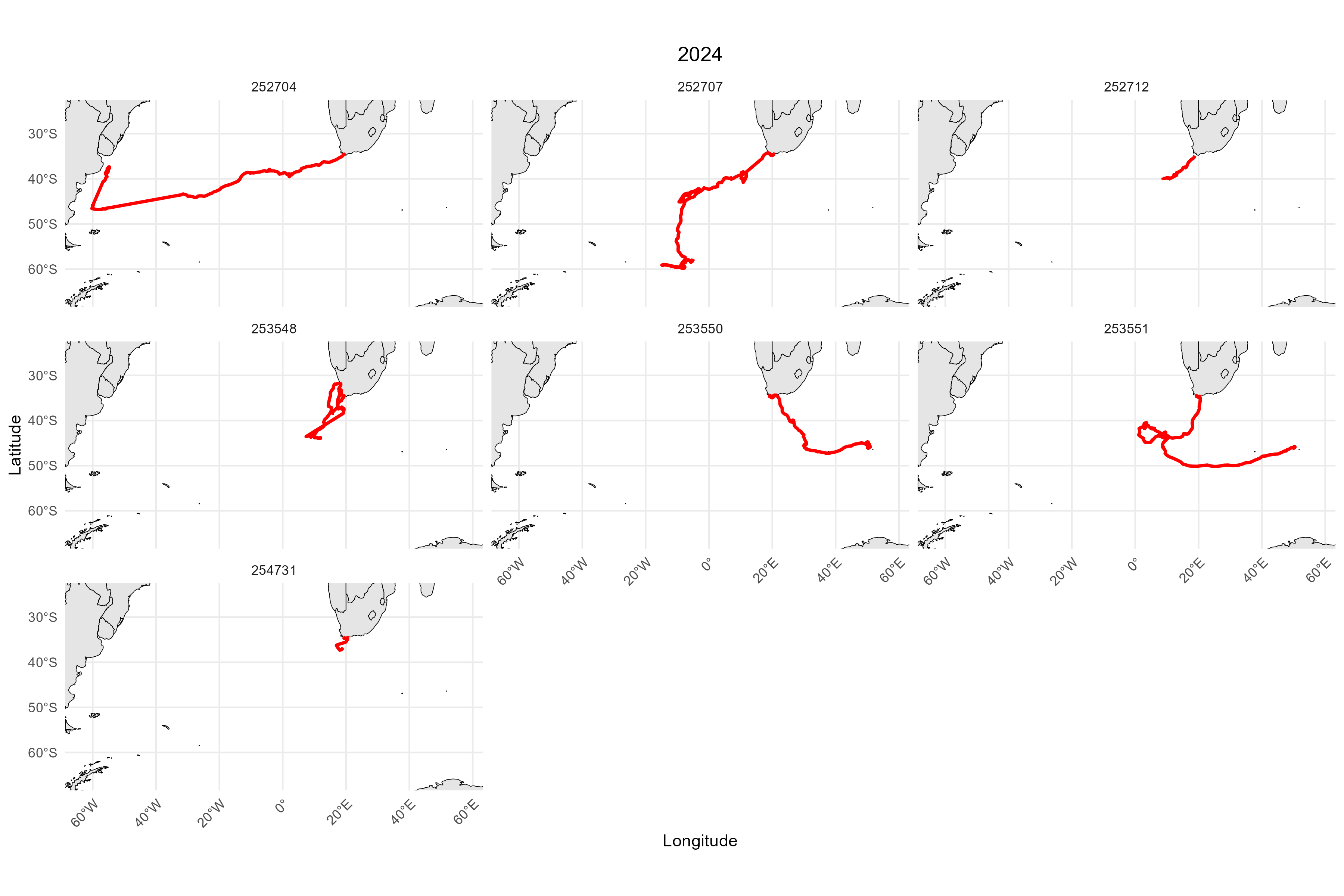

Supplement: Supplementary file 4 — Figure S4: Individual movement tracks of southern right whales ( Eubalaena australis ) deployed in Walker Bay, South Africa in 2024. Each panel shows the track of a tagged whale, faceted by ID. [file ECE3-16-e73975-s008.png]

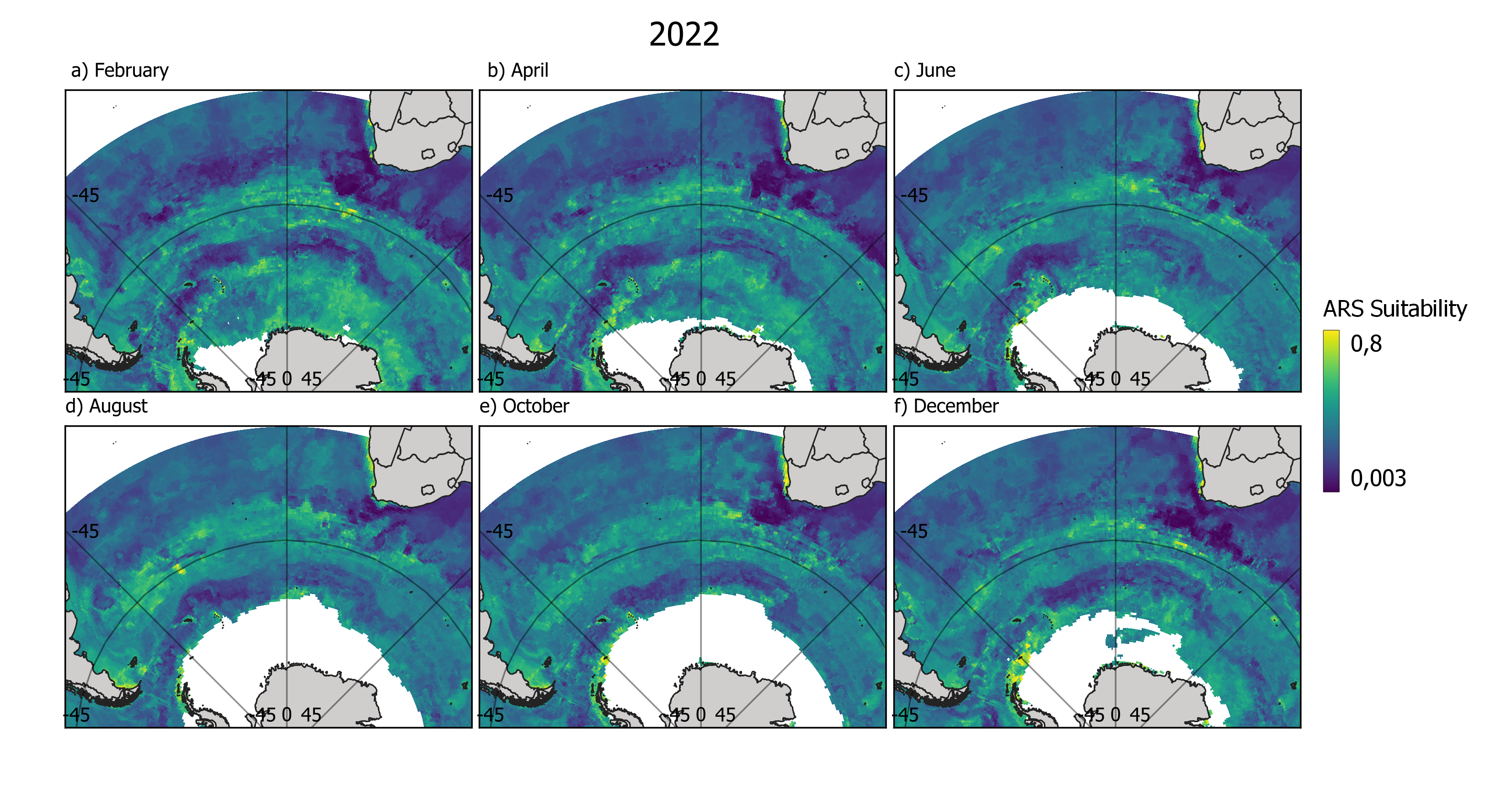

Supplement: Supplementary file 5 — Figure S5: Monthly habitat suitability predictions of area‐restricted search (ARS) behaviour based on random forest models trained using telemetry data from South African southern right whales tagged between 2021 and 2025. Predictions are shown for six representative months: (a) February, (b) April, (c) June, (d) August, (e) October, and (f) December from 2022. Foraging habitat suitability increases from blue to yellow. [file ECE3-16-e73975-s006.png]

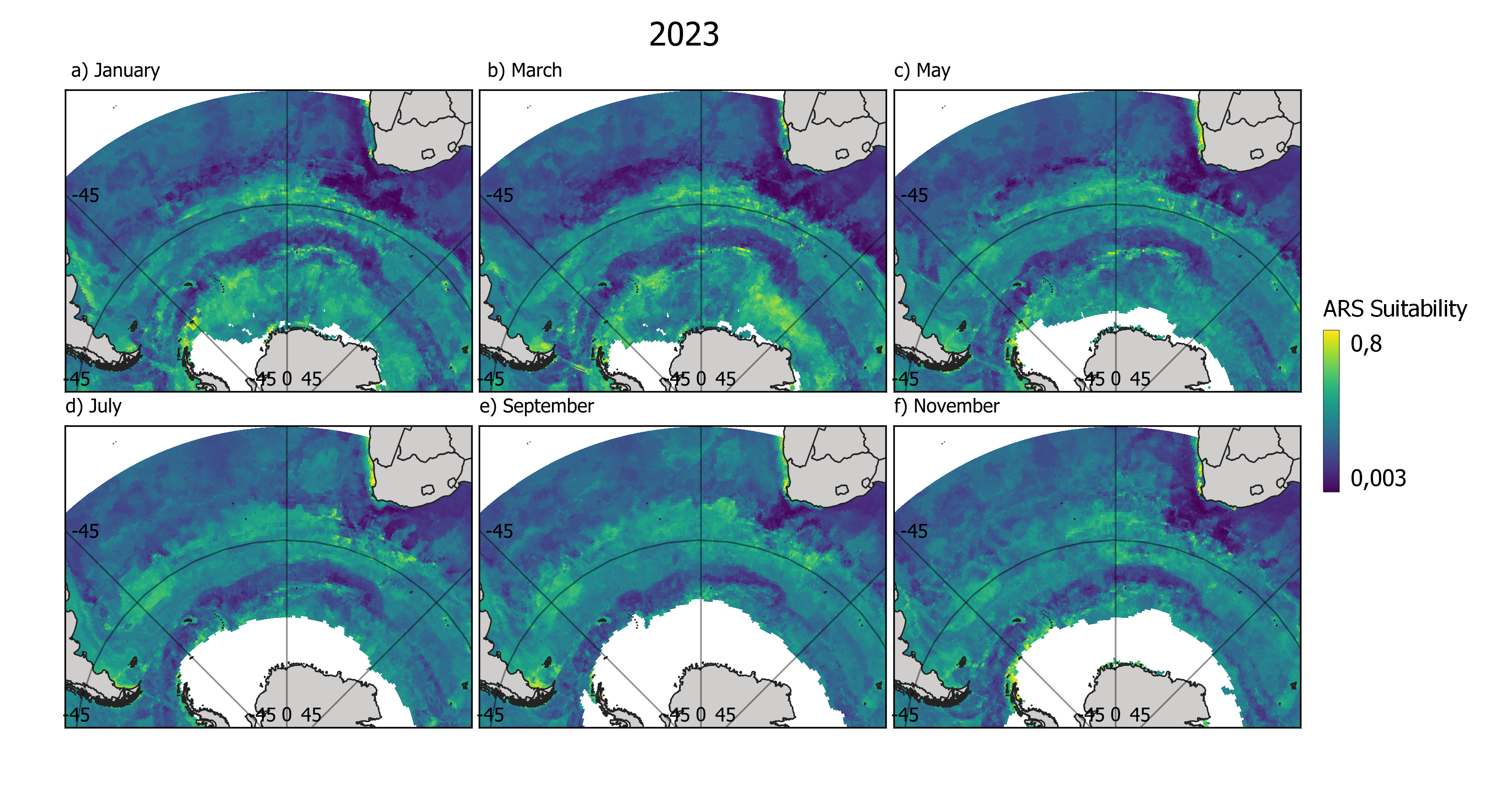

Supplement: Supplementary file 6 — Figure S6: Monthly habitat suitability predictions of area‐restricted search (ARS) behaviour based on random forest models trained using telemetry data from South African southern right whales tagged between 2021 and 2025. Predictions are shown for six representative months: (a) January, (b) March, (c) May, (d) July, (e) September, and (f) November from 2023. Foraging habitat suitability increases from blue to yellow. [file ECE3-16-e73975-s005.png]

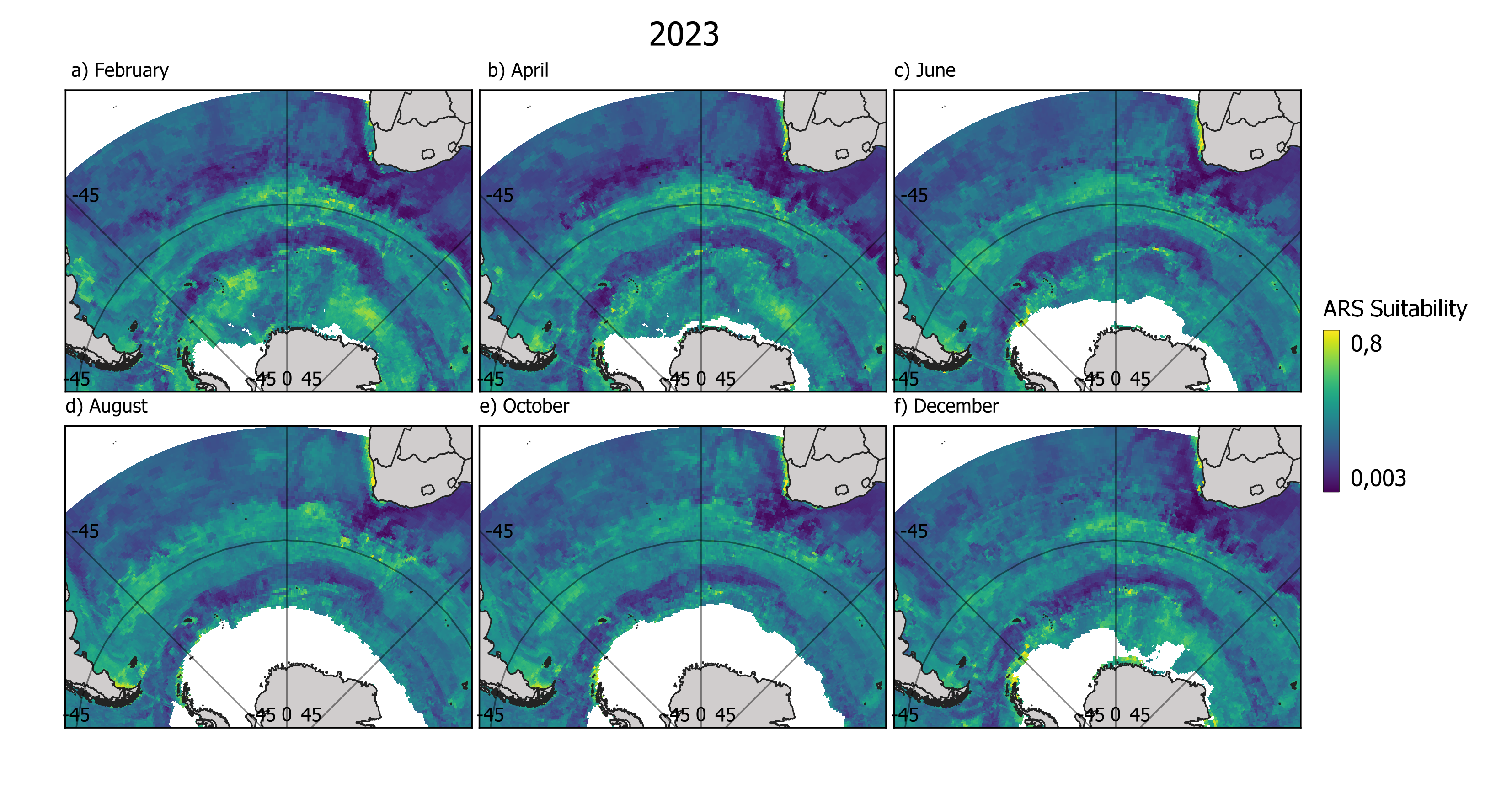

Supplement: Supplementary file 7 — Figure S7: Monthly habitat suitability predictions of area‐restricted search (ARS) behaviour based on random forest models trained using telemetry data from South African southern right whales tagged between 2021 and 2025. Predictions are shown for six representative months: (a) February, (b) April, (c) June, (d) August, (e) October, and (f) December from 2023. Foraging habitat suitability increases from blue to yellow. [file ECE3-16-e73975-s009.png]

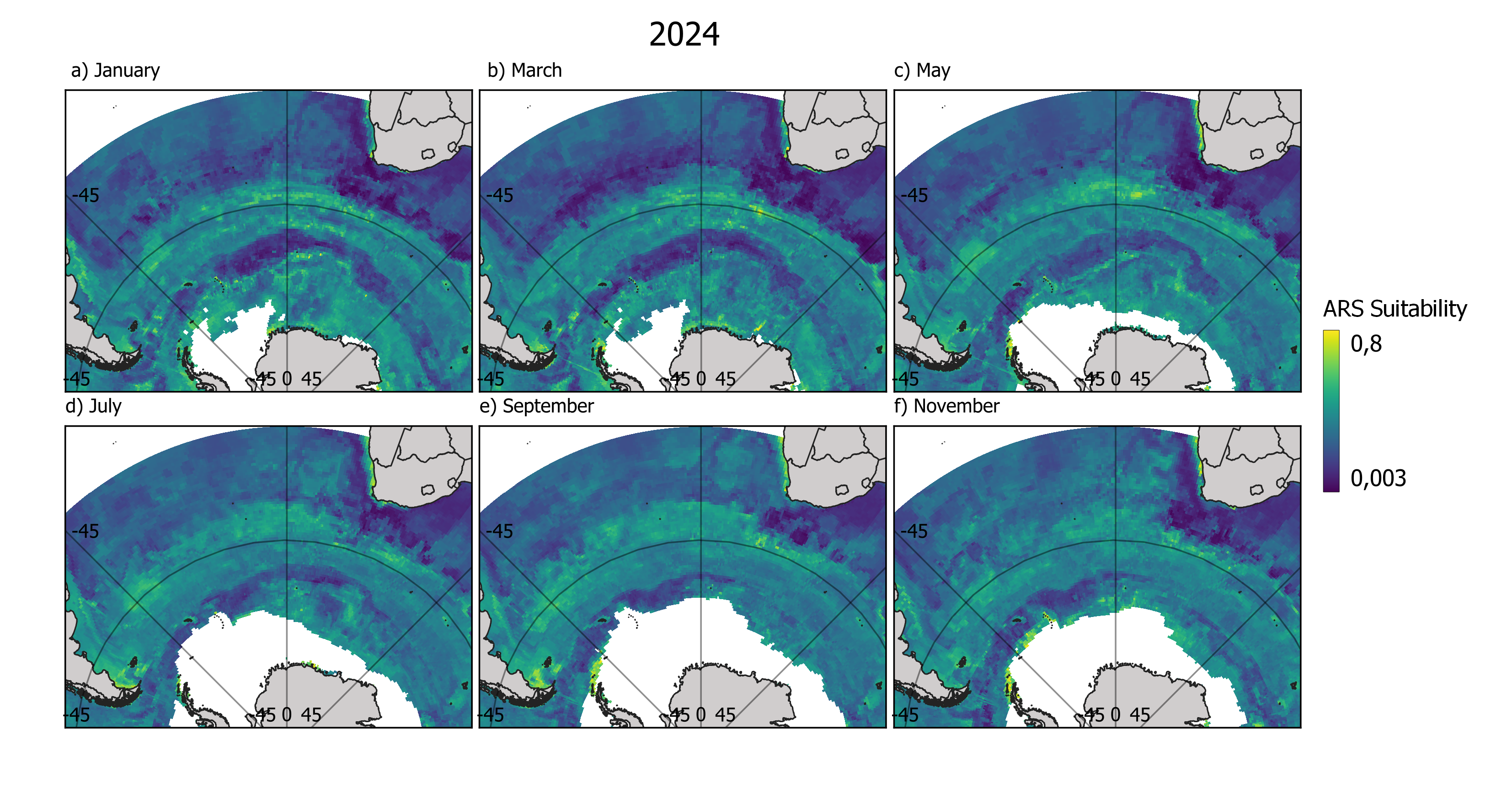

Supplement: Supplementary file 8 — Figure S8: Monthly habitat suitability predictions of area‐restricted search (ARS) behaviour based on random forest models trained using telemetry data from South African southern right whales tagged between 2021 and 2025. Predictions are shown for six representative months: (a) January, (b) March, (c) May, (d) July, (e) September, and (f) November from 2024. Foraging habitat suitability increases from blue to yellow. [file ECE3-16-e73975-s011.png]

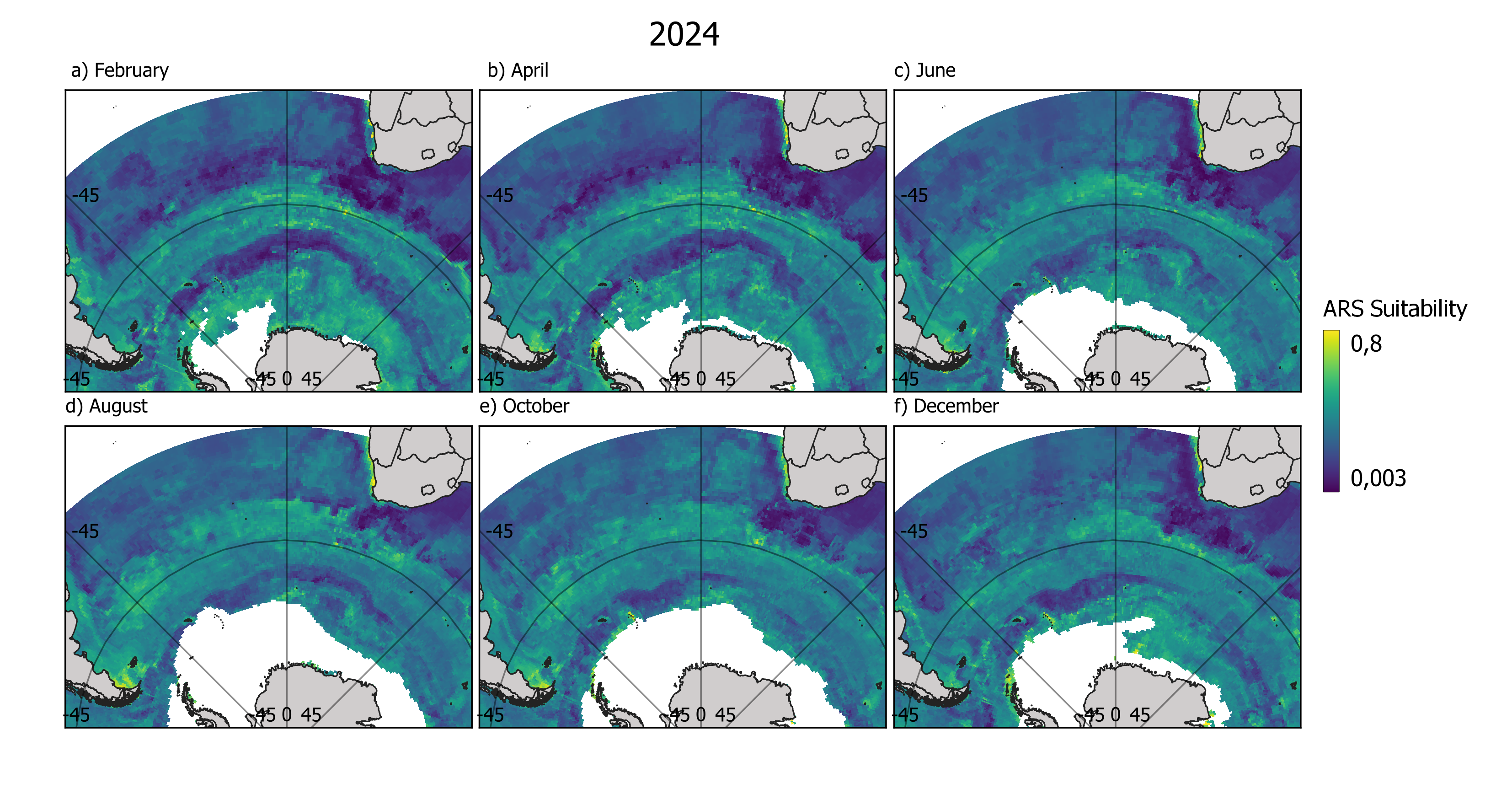

Supplement: Supplementary file 9 — Figure S9: Monthly habitat suitability predictions of area‐restricted search (ARS) behaviour based on random forest models trained using telemetry data from South African southern right whales tagged between 2021 and 2025. Predictions are shown for six representative months: (a) February, (b) April, (c) June, (d) August, (e) October, and (f) December from 2024. Foraging habitat suitability increases from blue to yellow. [file ECE3-16-e73975-s003.png]

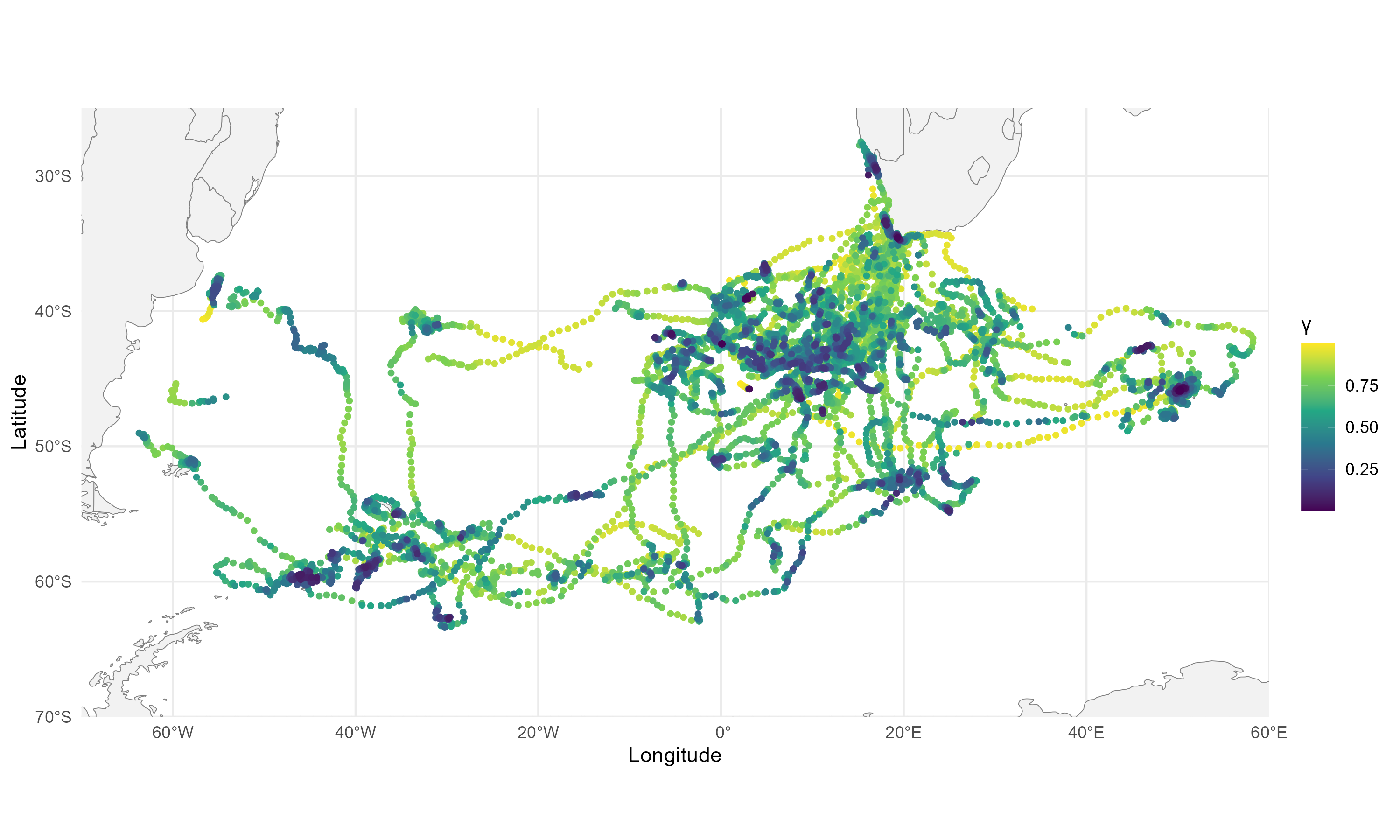

Supplement: Supplementary file 10 — Figure S10: Locations of satellite‐tracked Southern right whale coloured according to the estimated movement persistence parameter (γ) derived from the movement model. Lower values (purple/blue) indicate more tortuous movement associated with area‐restricted search or potential foraging behaviour, whereas higher values (green/yellow) indicate more directed movement consistent with transiting or migratory behaviour. [file ECE3-16-e73975-s001.png]
